# Spongenolactones A–C, Bioactive 5,5,6,6,5-Pentacyclic Spongian Diterpenes from the Red Sea Sponge *Spongia* sp.

**DOI:** 10.3390/md20080498

**Published:** 2022-08-01

**Authors:** Chi-Jen Tai, Atallah F. Ahmed, Chih-Hua Chao, Chia-Hung Yen, Tsong-Long Hwang, Fang-Rong Chang, Yusheng M. Huang, Jyh-Horng Sheu

**Affiliations:** 1 Doctoral Degree Program in Marine Biotechnology, National Sun Yat-sen University, Kaohsiung 80424, Taiwan; d035620001@nsysu.edu.tw; 2 Department of Pharmacognosy, College of Pharmacy, King Saud University, Riyadh 11451, Saudi Arabia; afahmed@ksu.edu.sa; 3 Department of Pharmacognosy, Faculty of Pharmacy, Mansoura University, Mansoura 35516, Egypt; 4 School of Pharmacy, China Medical University, Taichung 40604, Taiwan; chchao@mail.cmu.edu.tw; 5 Chinese Medicine Research and Development Center, China Medical University Hospital, Taichung 40604, Taiwan; 6 Graduate Institute of Natural Products, College of Pharmacy, Kaohsiung Medical University, Kaohsiung 80708, Taiwan; chyen@kmu.edu.tw (C.-H.Y.); aaronfrc@kmu.edu.tw (F.-R.C.); 7 National Natural Product Libraries and High-Throughput Screening Core Facility, Kaohsiung Medical University, Kaohsiung 80708, Taiwan; 8 Graduate Institute of Natural Products, College of Medicine, Chang Gung University, Taoyuan 333, Taiwan; htl@mail.cgu.edu.tw; 9 Research Center for Chinese Herbal Medicine, Graduate Institute of Healthy Industry Technology, College of Human Ecology, Chang Gung University of Science and Technology, Taoyuan 33303, Taiwan; 10 Department of Anesthesiology, Chang Gung Memorial Hospital, Taoyuan 333423, Taiwan; 11 Department of Marine Recreation, National Penghu University of Science and Technology, Magong, Penghu 88046, Taiwan; yusheng@gms.npu.edu.tw; 12 Tropical Island Sustainable Development Research Center, National Penghu University of Science and Technology, Magong, Penghu 88046, Taiwan; 13 Department of Marine Biotechnology and Resources, National Sun Yat-sen University, Kaohsiung 80424, Taiwan; 14 Department of Medical Research, China Medical University Hospital, China Medical University, Taichung 404333, Taiwan

**Keywords:** Red Sea sponge, *Spongia* sp., 5,5,6,6,5-pentacyclic spongian diterpenes, anti-inflammatory assay, antibacterial assay

## Abstract

Three new 5,5,6,6,5-pentacyclic spongian diterpenes, spongenolactones A–C (**1**–**3**), were isolated from a Red Sea sponge *Spongia* sp. The structures of the new metabolites were elucidated by extensive spectroscopic analysis and the absolute configurations of **1–3** were determined on the basis of comparison of the experimental circular dichroism (CD) and calculated electronic circular dichroism (ECD) spectra. Compounds **1**–**3** are the first 5,5,6,6,5-pentacyclic spongian diterpenes bearing an *β*-hydroxy group at C-1. These metabolites were assayed for their cytotoxic, antibacterial, and anti-inflammatory activities. All three compounds were found to exert inhibitory activity against superoxide anion generation in fMLF/CB-stimulated human neutrophils. Furthermore, **1** showed a higher activity against the growth of *Staphylococcus aureus* in comparison to **2**.

## 1. Introduction

**Figure 1:**
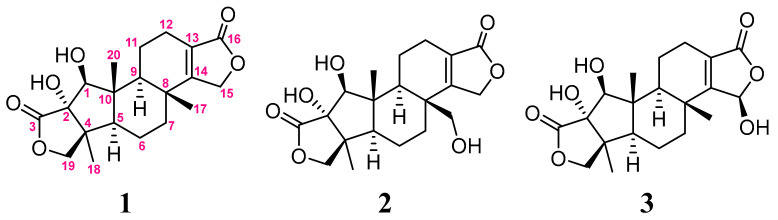
Structures of metabolites **1**–**3**.

Sponges of the genus *Spongia* have been proven to be rich sources of structurally diversified secondary metabolites [1,2]. A series of previous studies for the discovery of versatile molecular structures and bioactivities of compounds from sponges of the genus *Spongia* have been reported [3,4,5,6,7,8,9,10,11]. Our recent investigation on the secondary metabolites of a Red Sea sponge *Spongia* sp. has led to the isolation of a series of diverse new natural products, including a 5,5,6,6,5-pentacyclic diterpenoid, two furanotrinorsesquiterpenoid acids, a furanyl trinorsesterpenoid, a halogenated and oxygenated labdane, a highly oxygenated steroid, a steroid with the rare seven-membered lactone B ring, and an α,β-unsaturated fatty acid [12,13]. In our continuing effort to discover new metabolites from this sponge *Spongia* sp., we have further discovered three new diterpenes with 5,5,6,6,5-pentacyclic structures (5,6,6-tricarbocyclic ones with two five-membered lactones), spongenolactones A–C (**1**–**3**) (Figure 1). The molecular structures of **1**–**3** were established by detailed analysis of MS, IR, and NMR spectra (Supplementary Figures S1–S26), and by comparison of their NMR spectral data with those of structurally related known compounds. Further, the absolute configurations of **1**–**3** were determined by comparison of the experimental CD and calculated ECD spectra. Moreover, the cytotoxic activity of compounds **1**–**3** toward human hepatocellular carcinoma (HCC) Huh 7 cell line, their antibacterial activity against the growth of *Staphylococcus aureus*, and their anti-inflammatory activity toward the inhibition of the superoxide anion generation and elastase release in *N*-formyl-methionyl-leucyl phenylalanine/cytochalasin B (fMLF/CB)-induced human neutrophils, were also evaluated.

## 2. Results and Discussion

The sample of *Spongia* sp., collected off the Red Sea coast of Jeddah, Saudi Arabia, in 2016, was freeze-dried. The lyophilized sample (550 g) was chopped and extracted with EtOAc/MeOH/CH_2_Cl_2_. The crude extract was partitioned in water with CH_2_Cl_2_ to obtain the CH_2_Cl_2_ fraction (18.47 g), which was subjected to repeated column chromatography and high performance liquid chromatography (HPLC) to afford compounds **1** (2.4 mg), **2** (2.0 mg), and **3** (3.5 mg) (Figure 1).

**Figure 2:**
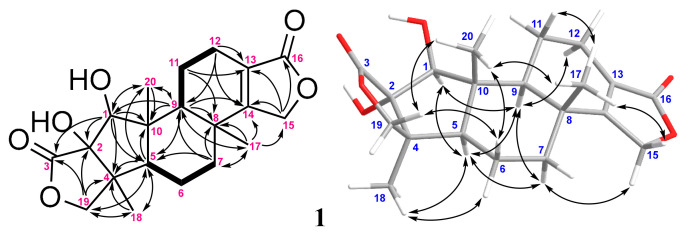
The selected COSY (▬), HMBC (→), and key NOESY (↔) correlations of **1**.

Compound **1** was isolated as a white powder. The HRESIMS of **1** (*m/z* 385.1623 [M + Na]^+^, calcd for C_20_H_26_O_6_Na, 385.1622, Supplementary Figure S1) revealed a molecular formula of C_20_H_2__6_O_6_, implying eight degrees of unsaturation. The IR spectrum of **1** showed the presence of hydroxy, carbonyl, and olefinic functionalities as revealed from the absorptions at 3444, 1747, 1683, and 1653 cm^−1^, respectively. The ^13^C NMR spectroscopic data of **1** showed 20 carbon signals (Table 1 and Supplementary Figure S3), which were assigned with the assistance of the DEPT spectrum into three methyls (δ_C_ 22.4, 22.4, and 15.6); six methylenes (δ_C_ 38.3, 22.2, 19.9, 18.6, including two oxygenated methylenes at δ_C_ 75.3 and 68.8); three methines (δ_C_ 56.1, 49.4, including one oxygenated methine at δ_C_ 91.2); and eight quaternary carbons (δ_C_ 183.3, 174.7, 172.2, 123.5, 90.2, 48.6, 47.4, and 38.0). In total, the NMR spectroscopic data of **1** (Table 1) displayed signals for an α-hydroxy-*γ*-lactone (δ_C_ 183.3 C, 90.2 C, 75.3 CH_2_, and 48.6 C; δ_H_ 4.40 and 3.83, each 1H, d, *J* = 10.0 Hz,) [12] and an unsaturated *γ*-lactone (δ_C_ 174.7 C, 172.2 C, 123.5 C, and 68.8 CH_2_; δ_H_ 4.82, 1H, tt, *J* = 17.0, 2.5 Hz and 4.51, 1H, dd, *J* = 17.0, 2.5 Hz) [14,15]. Further, the ^1^H–^1^H COSY experiment revealed the presence of two partial structures (Figure 2), which were connected by the HMBC correlations of **1** (Figure 2) to establish the 5,5,6,6,5-pentacyclic structure of **1** [3,4,12].

**Figure 3:**
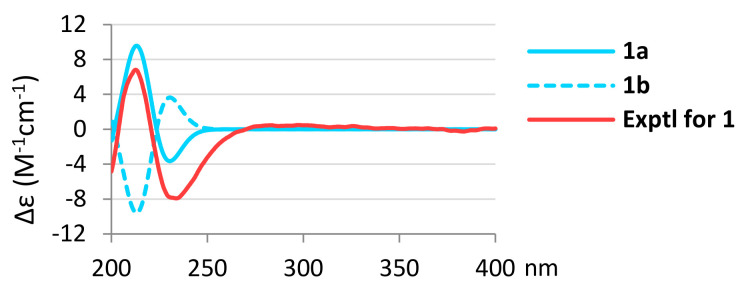
Calculated ECD curves of 1*S*,2*S*,4*S*,5*R*,8*R*,9*S*,10*R*-**1** (**1a**) and 1*R*,2*R*,4*R*,5*S*,8*S*,9*R*,10*S*-**1** (**1b**), and the experimental CD curve of **1**.

In the NOESY spectrum of **1**, the NOE interactions (Figure 2) of H_3_-20 with both H_3_-17 and one proton (δ_H_ 4.40) of H_2_-19, of H_3_-17 with one proton (δ_H_ 4.82) of H_2_-15 and of the proton at δ_H_ 4.40 of H_2_-19 with one proton (δ_H_ 1.75) at C-6 suggested that these protons should be positioned on the same plane, and were all assumed as β protons. By contrast, the correlations of H-6α (δ_H_ 1.60) with H_3_-18, of H_3_-18 with H-5, of H-5 with both H-7α (δ_H_ 1.22) and H-9, of H-7α with both H-9 and H-15α (δ_H_ 4.51); and of H-1 with both H-5 and H-9, revealed that these protons should be located on the same side, and were all assumed to be α-oriented. Further, the absolute configuration of **1** was determined by the comparison of the experimental CD and the calculated ECD spectra (Figure 3). The ECD curves of 1*S*,2*S*,4*S*,5*R*,8*R*,9*S*,10*R*-**1** (**1a**) and its enantiomer 1*R*,2*R*,4*R*,5*S*,8*S*,9*R*,10*S*-**1** (**1b**) were calculated at the CAM-B3LYP/6-311+G (d,p) level of theory, including an IEFPCM solvent model for MeOH, by the Gaussian 9.0 program [16,17]. The CD spectrum of **1** (Figure 3) showed positive and negative Cotton effects at 214.5 and 234.0 nm, respectively, which was found to be well consistent with the calculated ECD of **1a** (212.5 and 230.4 nm, Figure 3), and the absolute configuration of **1** was thus elucidated as 1*S*,2*R*,4*S*,5*R*,8*R*,9*S*, and 10*R*. Based on the above observations, the structure of **1** was identified as a new compound possessing the recently discovered 5,5,6,6,5-pentacyclic-based skeleton [3,4,12], but with a characteristic 1β-OH and named spongenolactone A. 

**Figure 4:**
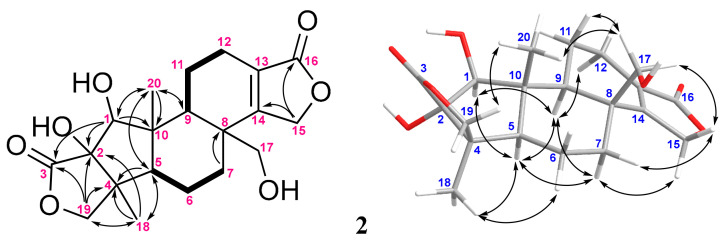
The selected COSY (▬), HMBC (→), and key NOESY (↔) correlations of **2**.

The ^1^H and ^13^C NMR data of metabolite **2** were very similar to those of **1** (Table 1), with the exception that the methyl group (δ_H_ 1.19, s, 3H and δ_C_ 22.4, CH_3_) at C-8 in **1** was oxidized to a hydroxymethyl (δ_H_ 3.67 and 4.01, both dd, *J*= 10.5, 4.5, 1H; δ_C_ 65.3, CH_2_) in **2**. The extensive analyses of COSY, HMBC, and NOESY 2D NMR experiments further confirmed that **2** is the 17-oxygenated derivative of **1** (Figure 4). 

**Figure 5:**
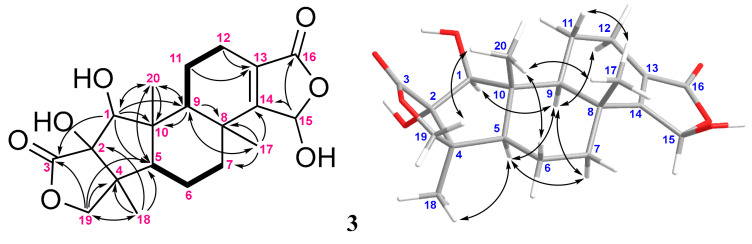
The selected COSY (▬), HMBC (→), and key NOESY (↔) correlations of **3**.

The ^1^H and ^13^C NMR data of compound **3** were also very similar to those of **1** (Table 1), except that the methylene group (δ_H_ 4.82, 1H, tt, *J* = 17.0, 2.5 Hz and 4.51, 1H, dd, *J* = 17.0, 2.5 Hz; δ_C_ 68.8, CH_2_) in the γ-lactone of **1** was converted to an acetal (δ_H_ 5.86, d, *J* = 2.0 Hz, 1H; δ_C_ 97.7, CH) in **3** (Figure 5). The detailed analyses of HMBC correlations (Figure 5 and Figure S24) confirmed that **3** is a 15-hydroxylated derivative of **1**. Furthermore, except for H-15 of **3**, which was observed not to exhibit any NOE interaction with other protons, the NOESY correlations (Figure 5) established the 1S*,2S*,4S*,5R*,8R*,9S*, and 10R* relative configuration of **3**. 

**Figure 6:**
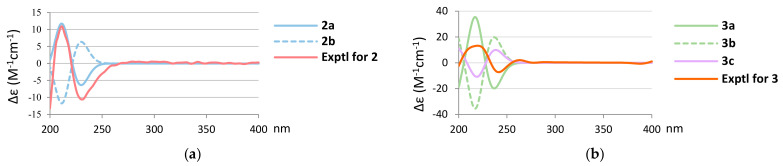
(**a**) Calculated ECD curves of 1*S*,2*S*,4*S*,5*R*,8*R*,9*S*,10*R*-**2** (**2a**), 1*R*,2*R*,4*R*,5*S*,8*S*,9*R*,10*S*-**2** (**2b**), and the experimental CD curve of **2**. (**b**) Calculated ECD curves of 1*S*,2*S*,4*S*,5*R*,8*R*,9*S*,10*R*,15*R*-**3** (**3a**), 1*R*,2*R*,4*R*,5*S*,8*S*,9*R*,10*S*,15*S*-**3** (**3b**), 1*S*,2*S*,4*S*,5*R*,8*R*,9*S*,10*R*,15*S*-**3** (**3c**), and the experimental CD curve of **3**.

Further, the absolute configuration of **2** was also elucidated by the comparison of the experimental CD and calculated ECD spectra (Figure 6a). The calculated ECD spectra of 1*S*,2*S*,4*S*,5*R*,8*R*,9*S*,10*R*-**2** (**2a**) and its enantiomer 1*R*,2*R*,4*R*,5*S*,8*S*,9*R*,10*S*-**2** (**2b**) were also obtained at the CAM-B3LYP/6-311+G (d,p) level of theory by the Gaussian 9.0 program. The CD spectrum of **2** (Figure 6a) showed positive and negative Cotton effects at 211.0 and 231.0 nm, respectively, which was found to match well with the calculated ECD of **2a**, which also showed corresponding positive and negative Cotton effects at 211.2 and 229.9 nm (Figure 6a). The absolute configuration of **2** was thus established as 1*S*,2*S*,4*S*,5*R*,8*R*,9*S*, and 10*R*. Moreover, the absolute stereochemistry of **3**, including the configuration of C-15, was elucidated to be that of **3a** (1*S*,2*S*,4*S*,5*R*,8*R*,9*S*,10*R*,15*R*) rather than that of the enantiomer **3b** (1*R*,2*R*,4*R*,5*S*,8*S*,9*R*,10*S*,15*S*) and the 15-epimer **3c** with a (1*S*,2*S*,4*S*,5*R*,8*R*,9*S*,10*R*,15*S*)-configuration (Figure 6b). Based on the above results, the structures of metabolites **2** and **3** were fully determined, and named spongenolactones B and C, respectively.

In order to discover the biological activity of new metabolites for future medicinal applications, **1**–**3** were tested for their cytotoxic, antibacterial, and anti-inflammatory activities. The cytotoxicity of **1**–**3** against HCC Huh7 cell line was evaluated by the resazurin assay [18,19], and none of the compounds showed notable activity against the growth of this cancer cell line. Furthermore, the assay for the growth inhibition of *S. aureus* showed that compound **1** exhibited 46%, 47%, and 93% inhibition at 50, 100, and 200 µM, respectively, while **2** displayed 24%, 42%, and 40% inhibition at 50, 100, and 200 µM, respectively.

The in vitro anti-inflammatory effects of **1**–**3** were also evaluated. At a concentration of 20 μM, compounds **1**–**3** displayed anti-inflammatory activity in suppressing the generation of superoxide anion (O_2_^−^**^·^**) and the elastase release, relative to the corresponding values of the control cells stimulated with fMLF/CB (Table 2) [20,21,22]. At 20 μM, **1**–**3** exhibited inhibitory activity against the generation of superoxide anion (61.0 ± 4.4%, 70.8 ± 4.8%, and 58.9 ± 6.0%, respectively) with IC_50_ values of 16.5 ± 1.6, 13.1 ± 1.3, and 17.4 ± 1.9 μM, respectively. Furthermore, **2** displayed an inhibitory activity against elastase release (52.2 ± 1.4%) at 20 μM with IC_50_ values of 18.6 ± 0.9 μM. Metabolites **1** and **3** also showed inhibitory activity against elastase release (49.8 ± 4.2% and 46.5 ± 6.2%, respectively).

## 3. Materials and Methods

### 3.1. General Experimental Procedures

Measurements of optical rotations, IR, and circular dichroisms spectra were performed on the JASCO P-1020 polarimeter, FT/IR-4100 infrared spectrophotometer (JASCO Corporation, Tokyo, Japan), and Jasco J-715 CD spectrometer, respectively. LRESIMS were measured on a Bruker APEX II (Bruker, Bremen, Germany) mass spectrometer, and HRESIMS were measured on the Bruker APEX II and the Impact HD Q-TOF mass spectrometers (both Bruker, Bremen, Germany). The NMR experiments were recorded on a Varian Unity INOVA 500 FT-NMR (Varian Inc., Palo Alto, CA, USA). The silica gel (40−63 μm, Merck, Billerica, MA, USA) and reversed-phase silica gel (RP-18, 40−63 μm, Merck, Darmstadt, Germany) were used for column chromatography. The thin-layer chromatography (TLC) analysis was carried out on aluminum plates coated with silica gel (Silica gel 60 F254, 100 μm, Merck, Darmstadt, Germany) or C18 gel (Silica gel 60 RP-18 F_254_s, 100 μm, Merck, Darmstadt, Germany). High-performance liquid chromatography (HPLC) was performed on a Hitachi L-2455 HPLC apparatus (Hitachi, Tokyo, Japan) with a Supelco C18 column (250 × 21.2 mm, 5 μm, Supelco, Bellefonte, PA, USA). 

### 3.2. Animal Material

The marine organism *Spongia* sp. was collected off the Red Sea coast of Jeddah, Saudi Arabia (21°22′11.08″ N, 39°06′56.62″ E) in March 2016. The biological sample (RSS-1) was stored at the Department of Pharmacognosy, College of Pharmacy, King Saud University, Saudi Arabia.

### 3.3. Extraction and Separation

The sample of *Spongia* sp. (550 g dry wt) was freeze-dried, chopped, and exhaustively extracted with EtOAc/MeOH/CH_2_Cl_2_ (1:1:0.5). The combined crude extract was suspended in water, and then partitioned in order with CH_2_Cl_2_, EtOAc, and *n*-BuOH to obtain CH_2_Cl_2_ (18.47 g), EtOAc (0.78 g), and *n*-BuOH (1.0 g) fractions, respectively. The CH_2_Cl_2_ fraction was chromatographed to yield 12 fractions (F1–F12) as described previously [13].

Fraction F7 (1.505 g, 75 % EtOAc/*n*-hexane) was separated by chromatography using an RP-18 column with the elution of H_2_O in MeOH (100% to 0%, stepwise) to afford eight subfractions (F7-1 to F7-8). Subfraction F7-3 (146.3 mg, 75 % MeOH/H_2_O) was further chromatographed to give ten subfractions (F7-3-1 to F7-3-10) by RP-18 HPLC (50 % MeOH/H_2_O). F7-3-4 (17.6 mg) was finally purified to isolate **3** (3.5 mg) by RP-18 HPLC (20% CH_3_CN/H_2_O). Moreover, F7-3-5 (22.7 mg) was purified to afford **1** (2.4 mg) and **2** (2.0 mg) by RP-18 HPLC (23% CH_3_CN/H_2_O). 

#### 3.3.1. Spongenolactone A (**1**)

White powder, [α]$\overset{25}{D}$ +37.0 (*c* = 0.24, CH_3_OH); IR (neat) ν_max_ 3444, 2922, 2851, 1747, 1683, and 1653 cm^−1^; ^1^H NMR and ^13^C data, see Table 1; HRESIMS *m/z* 385.1623 [M + Na]^+^ (calcd for C_20_H_26_O_6_Na, 385.1622).

#### 3.3.2. Spongenolactone B (**2**)

White powder, [α]$\overset{25}{D}$ +32.6 (*c* = 0.20, CH_3_OH); IR (neat) ν_max_ 3421, 2919, 2849, 1747, 1684, and 1655 cm^−1^; ^1^H NMR and ^13^C data, see Table 1; HRESIMS *m/z* 401.1573 [M + Na]^+^ (calcd for C_20_H_2__6_O_7_Na, 401.1571).

#### 3.3.3. Spongenolactone C (**3**)

White powder, [α]$\overset{25}{D}$ +39.0 (*c* = 0.35, CH_3_OH); IR (neat) ν_max_ 3445, 2919, 2849, 1747, 1683, and 1646 cm^−1^; ^1^H NMR and ^13^C data, see Table 1; HRESIMS *m/z* 379.1750 [M + H]^+^ (calcd for C_20_H_27_O_7_, 379.1751).

### 3.4. DFT and TD-DFT Calculations

The DFT approach at the B3LYP/6-31G (d,p) level of theory was applied to simulate the preliminary geometry optimization of conformers [16]. Then, the time-dependent DFT (TD-DFT) approach at the CAM-B3LYP/6-311+G(d,p) level of theory was used to simulate ECD spectra [16]. The integral equation formalism polarizable continuum (IEFPCM) solvent model for MeOH was used for the bulk solvent effect, with all programs calculated by the Gaussian 09 program [17]. The final ECD curves were transformed by GaussSum 2.2.5 and illustrated by Microsoft Excel.

### 3.5. Cytotoxicity Assay 

The cytotoxicity assay was performed using the methods described previously [18,19]. Huh7 cells were used in resazurin assay (Cayman Chemical) and treated with indicated concentrations (12.5, 50.0, and 200.0 µM) of compounds for 72 h. The DMSO control was assigned 100% of relative cell viability. The positive control, Sorafenib, inhibited the 52% growth of Huh7 cells at 12.5 µM.

### 3.6. Antibacterial Assay

The antibacterial assay was performed using the methods described previously [23]. The bacteria *S. aureus* were cultured in LB (Lysogeny broth) medium in the shaker incubator at 37 °C for 24 h. The bacterial culture was diluted to an absorbance of 0.04 at 600 nm using a sterile LB medium. Tested compounds (cpd) were then added to bacteria aliquots (100 μL/well of 96-well) with the concentrations at 50 μM, 100 μM, and 200 μM, respectively. Background control (1% DMSO in LB solution), positive control (1% DMSO in the diluted bacteria solution), and known drug control (tetracyclin; concentration is 0.5 µg/mL) were run on the same plate. The absorbance at 600 nm (A) was measured right after the testing compounds were added for the basal absorbance and after 16 h incubation at 37 °C. The percentage bacterial growth was calculated as follows: [(Acpd − Acpd_basal) − Abackground control]/[(Apositive control − Apositive control_basal) − Abackground control] × 100. 

### 3.7. Anti-Inflammatory Activity

The dextran sedimentation, Ficoll–Hypaque gradient centrifugation, and hypotonic lysis were used to enrich the neutrophils which were isolated from the blood of healthy adult volunteers, and these methods were described in a previous paper [22]. The neutrophils were incubated in Ca^2+^-free HBSS buffer (pH 7.4, ice-cold).

*Inhibition of Superoxide Anion Generation*

Neutrophils (6 × 10^5^ cells/mL) incubated in HBSS (with 0.6 mg/mL ferricytochrome *c* and 1 mM Ca^2+^, pH 7.4) at 37 °C were treated with the DMSO (as a control) or the tested compounds for 5 min. Neutrophils were activated by 100 nM fMLF for 10 min in the pretreatment of cytochalasin B (CB, 1 μg/mL) for 3 min (fMLF/CB). The generation of superoxide anion was spectrophotometrically measured at 550 nm (U-3010, Hitachi, Tokyo, Japan) [20,21]. LY294002 [2-(4-morpholinyl)-8-phenyl-1(4*H*)-benzopyran-4-one] was used as a positive control.

*Inhibition of Elastase Release*

Neutrophils (6 × 10^5^ cells/mL) incubated in HBSS (with 100 μM MeO-Suc-Ala-Ala- Pro-Val-*p*-nitroanilide and 1 mM Ca^2+^) at 37 °C were treated with DMSO (as a control) or the tested compound for 5 min. Neutrophils were activated with fMLF (100 nM)/CB (0.5 μg/mL) for 10 min. The generation of elastase release was spectrophotometrically measured at 405 nm (U-3010, Hitachi, Tokyo, Japan) [21].

## 4. Conclusions

Three new 5,5,6,6,5-pentacyclic spongian diterpenes, spongenolactones A–C (**1**–**3**), were isolated from a Red Sea sponge, *Spongia* sp. These metabolites are the first 5,5,6,6,5-pentacyclic spongian diterpenes bearing a *β*-hydroxy group at C-1. In our previous chemical study of the same organism, we also discovered a compound of the same skeleton, 17-dehydroxysponalactone, which was found to potently reduce the superoxide anion generation and elastase release [12]. Compounds **1**–**3** exhibited inhibitory activity against the generation of superoxide anion, and **2** also displayed inhibitory activity against elastase release. Furthermore, **1** showed significant inhibition on the growth of *S. aureus*.

## Figures and Tables

**Table 1:** ^13^C and ^1^H NMR data for compounds **1**–**3**^a.^.

	1		2		3	
Position	δ_H_	δ_C_	δ_H_	δ_C_	δ_H_	δ_C_
1	3.86, br s	91.2, CH	3.84 br s	91.1, CH	3.83, br s	91.2, CH_2_
2	−	90.2, C	−	90.1, C	−	90.4, C
3	−	183.3, C	−	182.4, C	−	183.3, C
4	−	48.6, C	−	48.6, C	−	48.7, C
5	2.00, dd (13.0, 1.5) ^b^	56.1, CH	2.01, m	56.2, CH	1.96, m	56.1, CH
6α	1.60, m	18.6, CH_2_	1.57 m	18.4, CH_2_	1.61, m	18.4, CH_2_
6β	1.75, m		1.71 dd (13.0, 3.0)		1.74, m	
7α	1.22, m	38.3, CH_2_	1.03 dt (13.0, 3.5)	33.2, CH_2_	1.14, m	37.8, CH_2_
7β	1.81, m		2.23 m		1.98, m	
8	−	38.0, C	−	44.2, C	−	38.1, C
9	1.88, dd (12.0, 1.0)	49.4, CH	1.93, dd (12.0, 2.0)	49.9, CH	1.74, m	50.0, CH
10	−	47.4, C	−	47.1, C	−	47.5, C
11α	1.79, m	19.9, CH_2_	1.76, m	19.5, CH_2_	1.77, m	19.8, CH_2_
11β	1.65, m		1.82, m		1.66, m	
12α	2.02, m	22.2, CH_2_	2.04 m	22.2, CH_2_	2.03, m	22.1, CH_2_
12β	2.22, m		2.24 m		2.26, m	
13	−	123.5, C	−	124.9, C	−	127.8, C
14	−	172.2, C	−	170.5, C	−	169.0, C
15α	4.51, dd (17.0, 2.5)	68.8, CH_2_	4.53, dd (17.0, 2.5)	72.4, CH_2_	5.86, d (2.0)	97.7, CH
15β	4.82, tt (17.0, 2.5)		4.96, tt (17.0, 2.5)			
16	−	174.7, C	−	174.8, C	−	172.9, C
17	1.19, s	22.4, CH_3_	3.67, dd (10.5, 4.5)	65.3, CH_2_	1.26, s	21.0, CH_3_
			4.01, dd (10.5, 4.5)			
18	1.13, s	22.4, CH_3_	1.12, s	22.4, CH_3_	1.14, s	22.4, CH_3_
19α	3.83, d (10.0)	75.3, CH_2_	3.82, d (10.0)	75.3, CH_2_	3.83, d (10.0)	75.4, CH_2_
19β	4.40, d (10.0)		4.40, d (10.0)		4.40, d (10.0)	
20	0.80, s	15.6, CH_3_	0.76, s	15.6, CH_3_	0.80, s	15.8, CH_3_

^a.^^13^C and ^1^H spectra recorded at 125 and 500 MHz in acetone-*d*_6_; ^b.^*J* values (Hz) in parentheses.

**Table 2:** Inhibitory effects of compounds **1**–**3** on superoxide anion generation and elastase release by human neutrophils.

Compound	Superoxide Anion		Elastase Release	
	IC_50_ (μM) ^a^	Inh% ^b^	IC_50_ (μM)	Inh%
**1**	16.5 ± 1.6	61.0 ± 4.4 ***	>20	49.8 ± 4.2 ***
**2**	13.1 ± 1.3	70.8 ± 4.8 ***	18.6 ± 0.9	52.2 ± 1.4 ***
**3**	17.4 ± 1.9	58.9 ± 6.0 ***	>20	46.5 ± 6.2 **
LY294002	1.9 ± 0.8	88.7 ± 1.5 ***	2.9 ± 0.1	79.5 ± 2.0 ***

Results are presented as mean ± S.E.M. (*n* = 3). ** *p* < 0.01, *** *p* < 0.001 compared with the control value (DMSO). ^a^ Concentration required for 50% inhibition (IC50). ^b^ Percentage of inhibition (Inh %) at 20 μM.

## Data Availability

Data of the present study are available in the article and Supplementary Materials.

## Supplementary Materials

The following supporting information can be downloaded at: https://www.mdpi.com/article/10.3390/md20080498/s1, Table S1. The cartesian coordinates of conformer **1**, Table S2. The cartesian coordinates of conformer **2**, Table S3. The cartesian coordinates of conformer **3**, Table S4. The cartesian coordinates of conformer **3c**, Table S5. Cytotoxicity (ED_50_ μg/mL) of compounds **1**–**3**, Figures S1–S26: HRESIMS spectra and 1D and 2D NMR spectra of compounds **1**–**3**.
