# Supplementary material for: Spongenolactones A–C, Bioactive 5,5,6,6,5-Pentacyclic Spongian Diterpenes from the Red Sea Sponge Spongia sp
Source: Mar Drugs. 2022 Aug 1;20(8):498. doi: 10.3390/md20080498 (PMC9410434; doi:10.3390/md20080498)
Supplement: Supplementary file 1 [file marinedrugs-20-00498-s001.zip › marinedrugs-1814868-supplementary.pdf]

Spongenolactones A–C, Bioactive 5,5,6,6,5-Pentacyclic Spongian Diterpenes from the Red Sea Sponge *Spongia* sp.

Chi-Jen Tai [1], Atallah F. Ahmed [2,3], Chih-Hua Chao [4,5], Chia-Hung Yen [6,7], Tsong-Long Hwang [8,9,10], Fang-Rong Chang [6], Yusheng M. Huang [11,12] and Jyh-Horng Sheu [1,6,13,14,*]

1 Doctoral Degree Program in Marine Biotechnology, National Sun Yat-sen University, Kaohsiung 80424, Taiwan; d035620001@nsysu.edu.tw
2 Department of Pharmacognosy, College of Pharmacy, King Saud University, Riyadh 11451, Saudi Arabia; afahmed@ksu.edu.sa
3 Department of Pharmacognosy, Faculty of Pharmacy, Mansoura University, Mansoura 35516, Egypt
4 School of Pharmacy, China Medical University, Taichung 40604, Taiwan; chchao@mail.cmu.edu.tw
5 Chinese Medicine Research and Development Center, China Medical University Hospital, Taichung 40604, Taiwan
6 Graduate Institute of Natural Products, College of Pharmacy, Kaohsiung Medical University, Kaohsiung 80708, Taiwan; chyen@kmu.edu.tw (C.-H.Y.), aaronfrc@kmu.edu.tw (F.-R.C.)
7 National Natural Product Libraries and High-Throughput Screening Core Facility, Kaohsiung Medical University, Kaohsiung 80708, Taiwan
8 Graduate Institute of Natural Products, College of Medicine, Chang Gung University, Taoyuan 333, Taiwan; htl@mail.cgu.edu.tw
9 Research Center for Chinese Herbal Medicine, Graduate Institute of Healthy Industry Technology, College of Human Ecology, Chang Gung University of Science and Technology, Taoyuan 33303, Taiwan
10 Department of Anesthesiology, Chang Gung Memorial Hospital, Taoyuan 333423, Taiwan
11 Department of Marine Recreation, National Penghu University of Science and Technology, Magong, Penghu 88046, Taiwan; yusheng@gms.npu.edu.tw
12 Tropical Island Sustainable Development Research Center, National Penghu University of Science and Technology, Magong, Penghu 88046, Taiwan.
13 Department of Marine Biotechnology and Resources, National Sun Yat-sen University, Kaohsiung 80424, Taiwan.
14 Department of Medical Research, China Medical University Hospital, China Medical University, Taichung 404333, Taiwan.
* Correspondence: sheu@mail.nsysu.edu.tw; Tel.: +886-7-525-2000 (ext. 5030); Fax: +886-7-525-5020.

Contents

Table S1. The cartesian coordinates of conformer **1**. 8

Table S2. The cartesian coordinates of conformer **2**. 17

Table S3. The cartesian coordinates of conformer **3**. 22

Table S4. The cartesian coordinates of conformer **3c**. 23

Table S5. Cytotoxicity (ED_{50} μg/mL) of compounds **1**−**3**. 24

Figure S1. HRESIMS spectrum of **1**. 3

Figure S2. ^{1}H NMR spectrum of **1**. 3

Figure S3. ^{13}C NMR spectrum of **1**. 4

Figure S4. DEPT spectrum of **1**. 4

Figure S5. HSQC spectrum of **1** 5

Figure S6. ^{1}H−^{1}H COSY spectrum of **1**. 5

Figure S7. HMBC spectrum of **1**. 6

Figure S8. NOESY spectrum of **1**. 6

Figure S9. The partial NOESY spectrum of **1**. 7

Figure S10. HRESIMS spectrum of **2**. 12

Figure S11. ^{1}H NMR spectrum of **2**. 12

Figure S12. ^{13}C NMR spectrum of **2**. 13

Figure S13. DEPT spectrum of **2**. 13

Figure S14. HSQC spectrum of **2**. 14

Figure S15. ^{1}H−^{1}H COSY spectrum of **2**. 14

Figure S16. HMBC spectrum of **2**. 15

Figure S17. NOESY spectrum of **2**. 15

Figure S18. The partial NOESY spectrum of **2**. 16

Figure S19. HRESIMS spectrum of **3**. 18

Figure S20. ^{1}H NMR spectrum of **3**. 18

Figure S21. ^{13}C NMR spectrum of **3**. 19

Figure S22. HSQC spectrum of **3**. 19

Figure S23. ^{1}H–^{1}H COSY spectrum of **3**. 20

Figure S24. HMBC spectrum of **3**. 20

Figure S25. NOESY spectrum of **3**. 21

Figure S26. The partial NOESY spectrum of **3**. 21

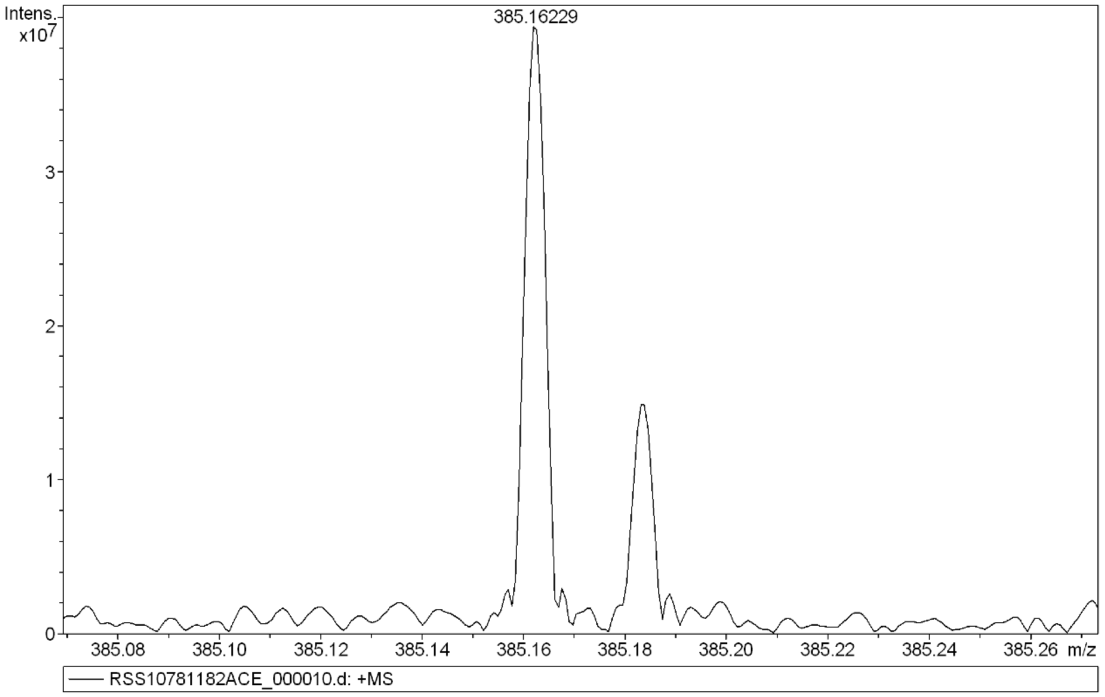

Meas. m/z	#	Formula	Score	m/z	err [mDa]	err [ppm]	mSigma	rdb	e⁻ Conf	N-Rule
385.16229	1	C 20 H 26 Na O 6	100.00	385.16216	-0.13	-0.33	20.9	7.5	even	ok

Figure S1. HRESIMS spectrum of **1**.

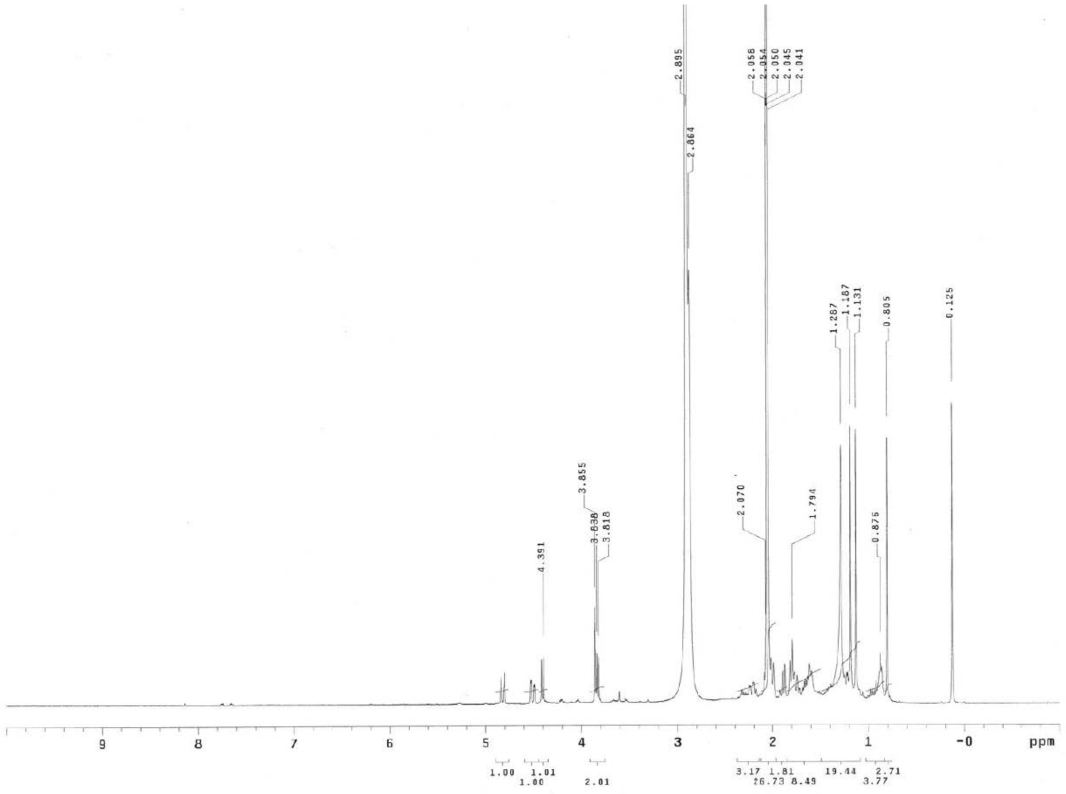

Figure S2. ^{1}H NMR spectrum of **1**.

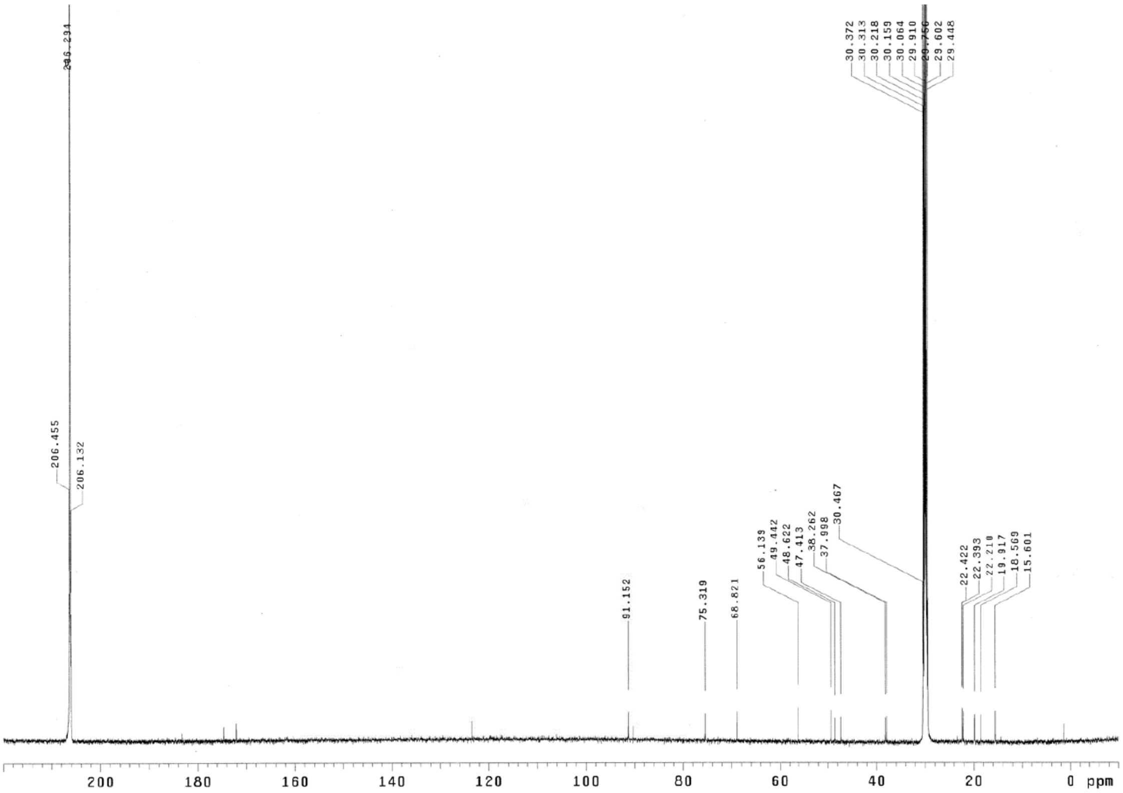

Figure S3.^{13}C NMR spectrum of **1**.

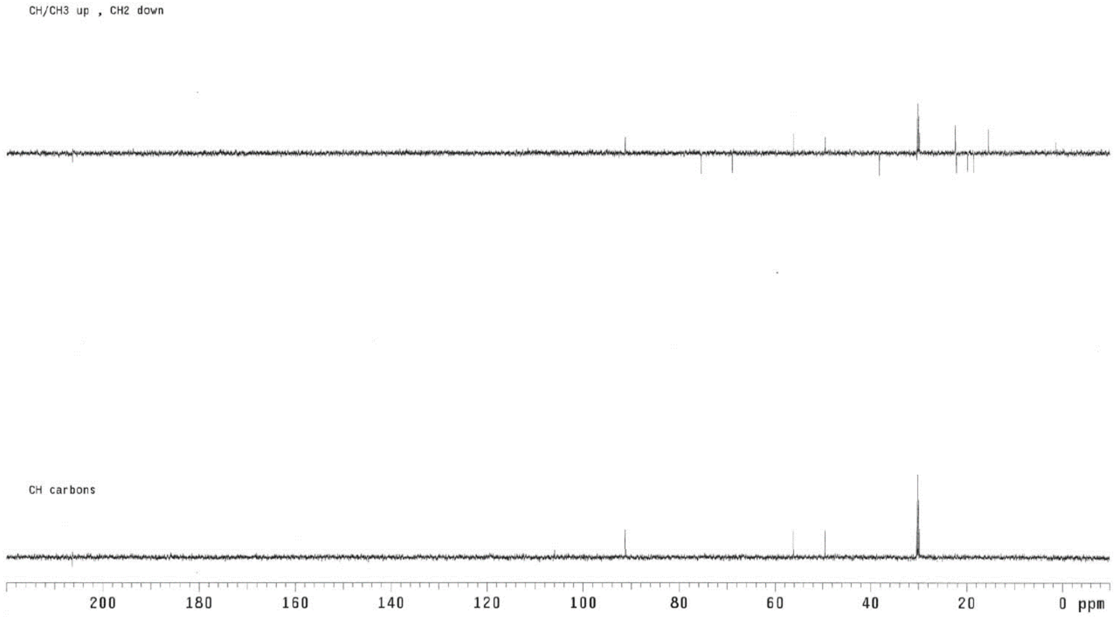

Figure S4. DEPT spectrum of **1**.

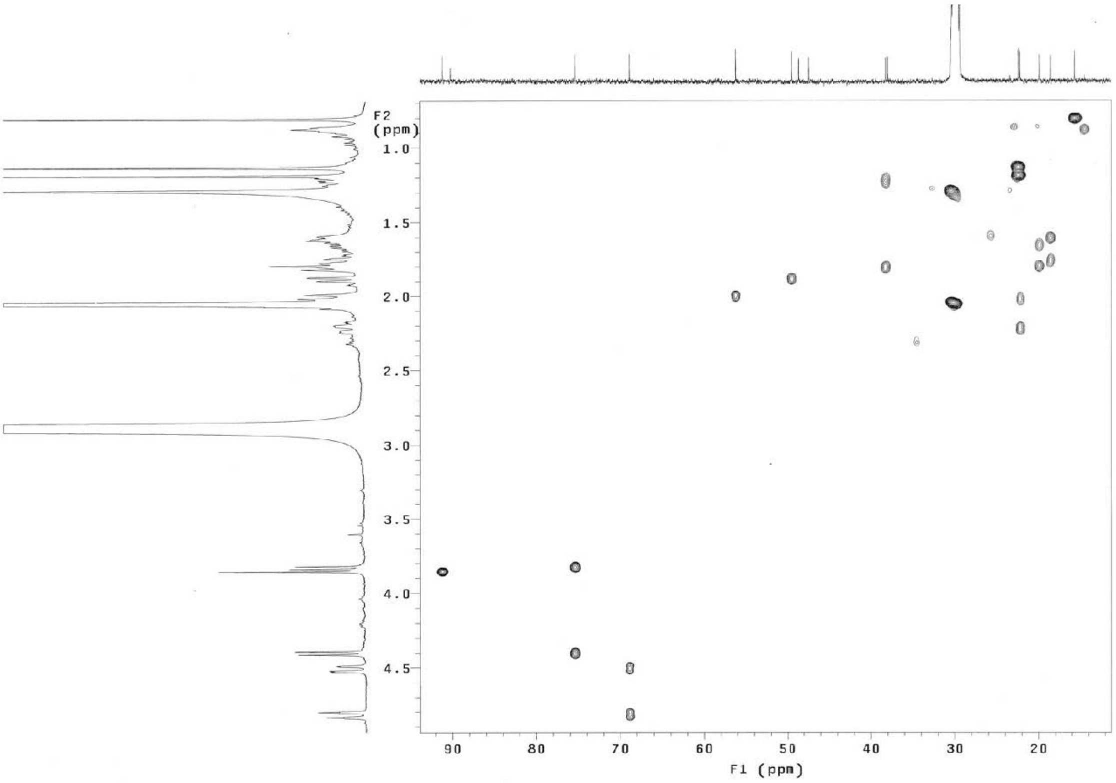

Figure S5. HSQC spectrum of **1**

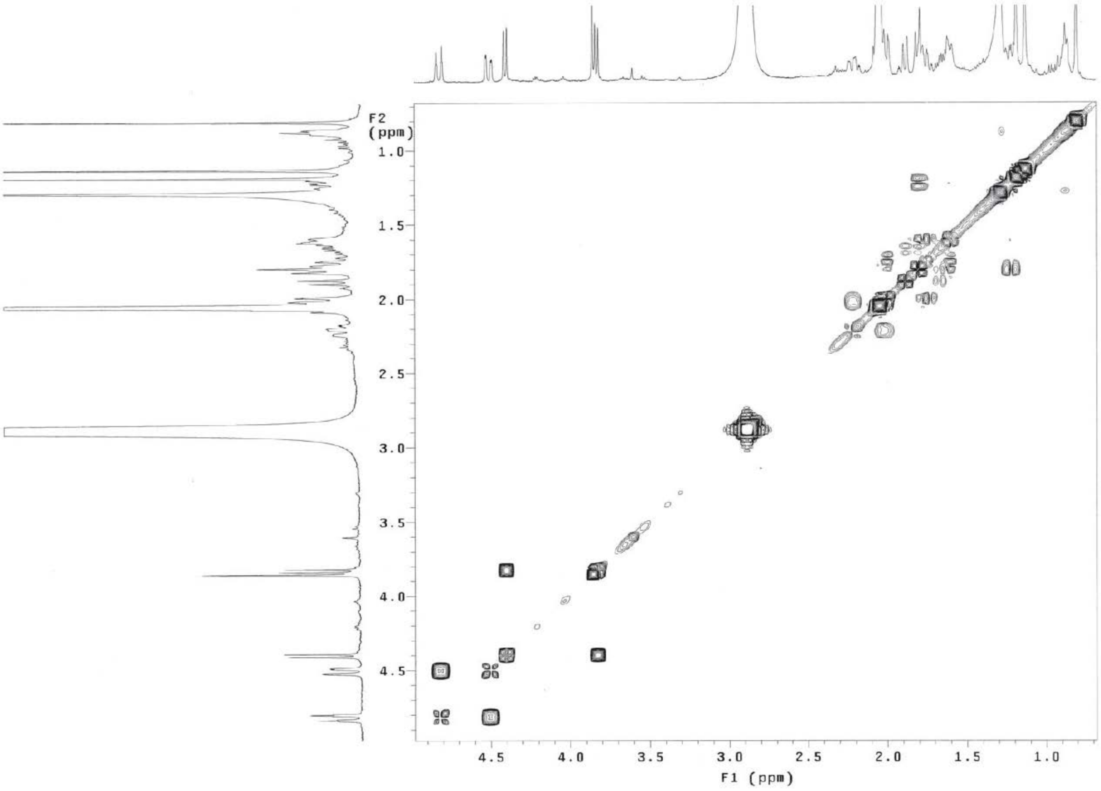

Figure S6. $^{1}H-^{1}H$ COSY spectrum of **1**.

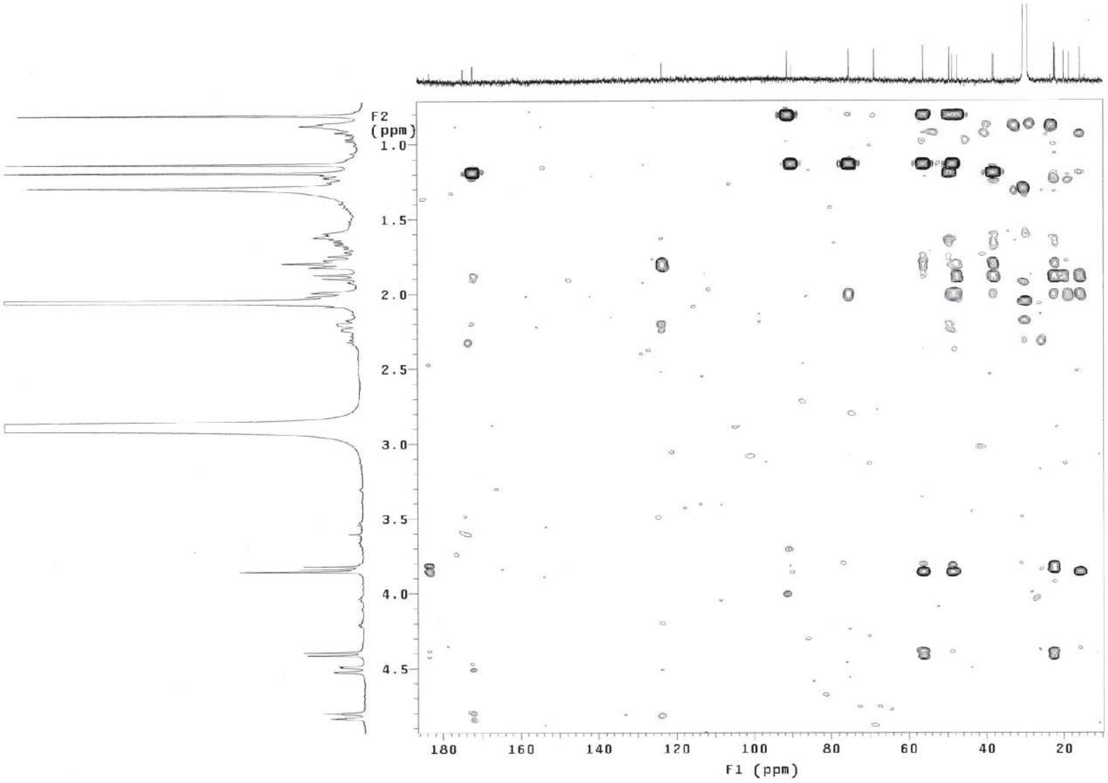

Figure S7. HMBC spectrum of **1**.

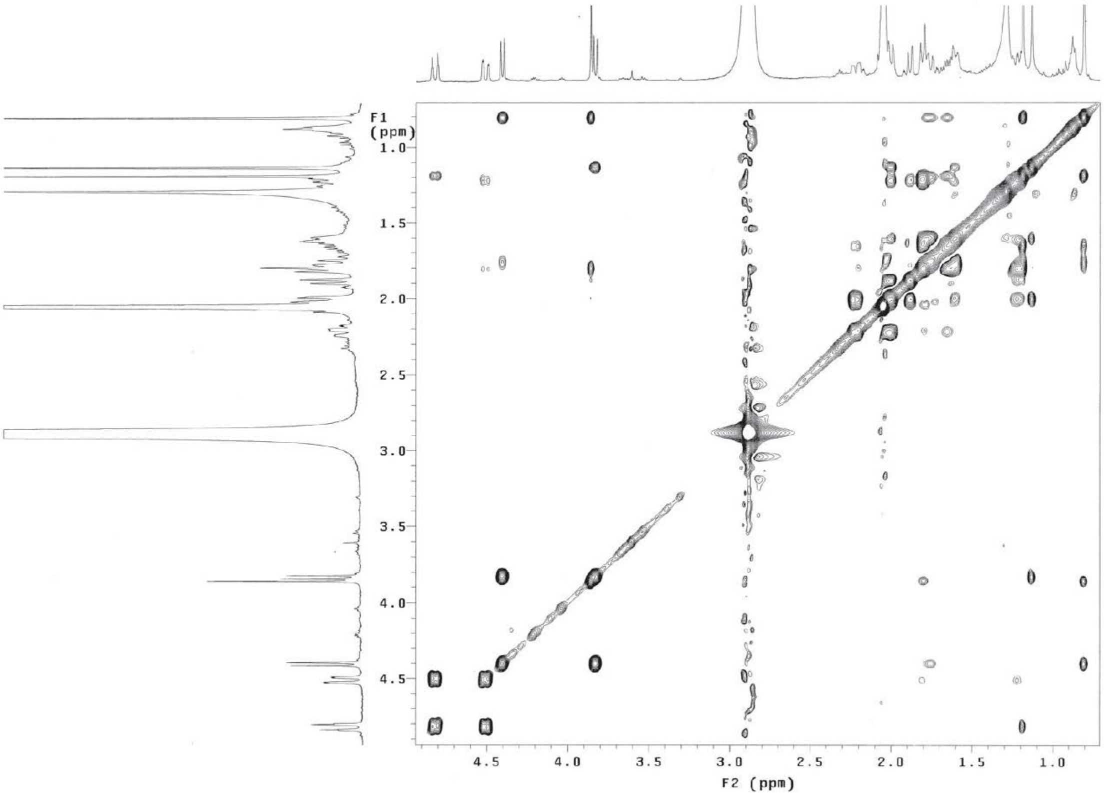

Figure S8. NOESY spectrum of **1**.

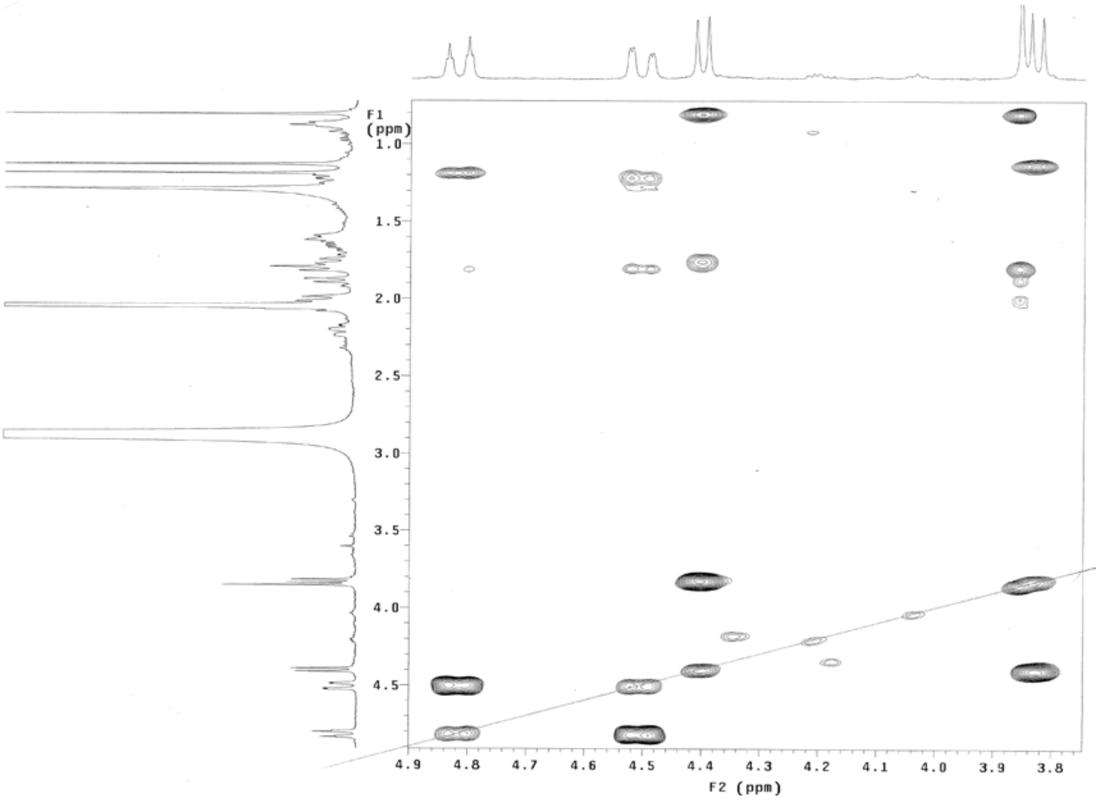

Figure S9. The partial NOESY spectrum of **1**.

Table S1. The cartesian coordinates of conformer **1**.

C	0.70248400	-0.20906500	0.39088000	H	0.70787800	0.89531800	-1.41518100
C	1.08764200	1.07733100	-0.39752400	H	0.56607200	2.55719300	1.12496100
C	0.34766600	2.32798100	0.07582700	H	0.65995800	3.20459500	-0.50263700
C	-1.16613600	2.10892300	-0.13621200	H	-1.34947600	2.04286600	-1.21644500
C	-1.70961500	0.82182500	0.54934800	H	-1.72536000	2.97987400	0.22537700
C	-0.82074900	-0.42075200	0.14823000	H	-0.91410100	-0.46910500	-0.94747700
C	-3.10126100	0.48946300	0.05290500	H	-2.62607300	-2.47184100	-0.99677300
C	-3.56092700	-0.75516100	-0.16189400	H	-3.35962000	-2.75732800	0.56675400
C	-2.78005000	-2.02344400	-0.00535500	H	-1.55539500	-1.70656900	1.75543900
C	-1.42113600	-1.74203200	0.66903700	H	-0.72921200	-2.56452400	0.46997100
C	-4.20689500	1.46648600	-0.24005100	H	-4.50476800	2.06293900	0.62945400
O	-5.32637000	0.65424300	-0.64161300	H	-3.96528700	2.15355200	-1.05846600
C	-4.96198700	-0.66941300	-0.60553300	H	2.13838300	-0.40151900	2.05460700
C	1.07604500	-0.21822200	1.89294700	H	0.54918200	-1.02370700	2.40873900
C	-1.85118700	1.08787500	2.07557200	H	0.82245200	0.71474800	2.39497600
O	-5.74056100	-1.55230600	-0.90624000	H	-2.04009600	0.17181600	2.63918000
C	1.59003100	-1.23133600	-0.36059600	H	-2.69381000	1.76255800	2.25616800
C	2.91957800	-0.47144500	-0.74354200	H	-0.96721900	1.57050500	2.49276000
C	2.62394900	1.05578000	-0.59617800	H	1.12881500	-1.46609700	-1.33156700
C	4.06363000	-0.73607100	0.22841000	H	2.85962600	1.72675800	1.52320300
O	4.28090100	0.31324500	1.03498300	H	4.15276000	2.27366500	0.44069400
C	3.46048500	1.45714200	0.65514900	H	2.37846100	-2.99949100	-0.10258600
O	1.78866500	-2.41481900	0.39439200	H	3.97500100	-1.58902300	-1.92499400
O	3.35282400	-0.85279300	-2.04197200	H	4.14373400	1.77174300	-1.98492900
O	4.73982600	-1.74336600	0.25177300	H	2.86645200	2.94644100	-1.63272300
C	3.07080900	1.88494500	-1.80588100	H	2.54510100	1.57614700	-2.71233700

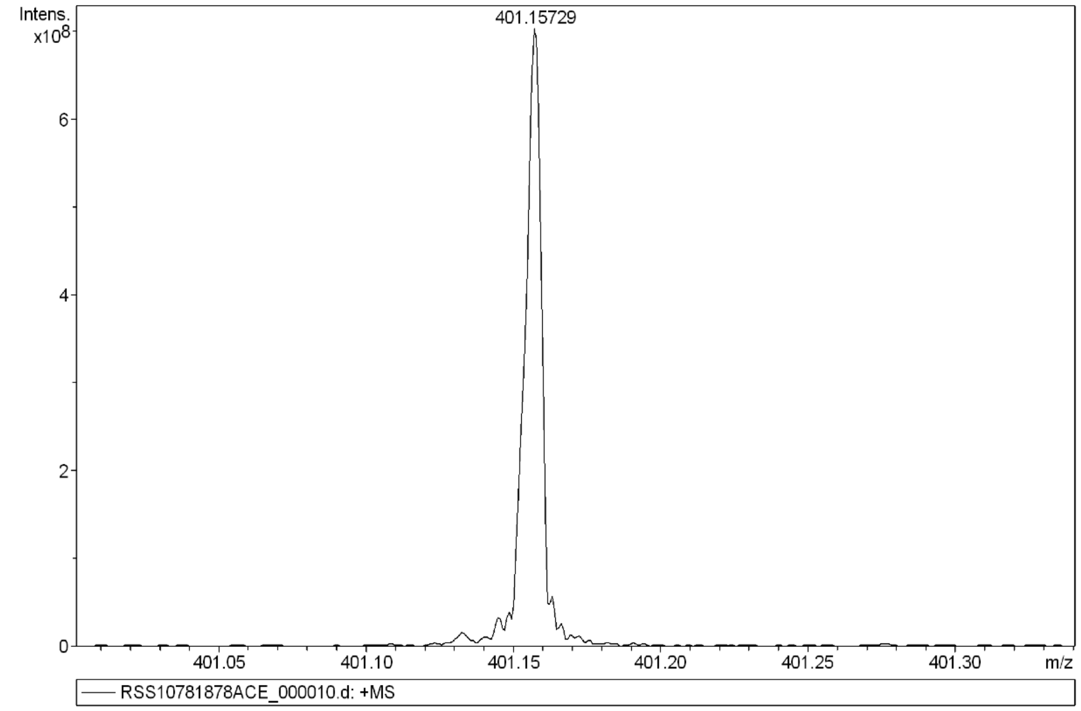

Meas. m/z	#	Formula	Score	m/z	err [mDa]	err [ppm]	mSigma	rdb	e⁻ Conf	N-Rule
401.15729	1	C 20 H 26 Na O 7	100.00	401.15707	-0.22	-0.54	11.1	7.5	even	ok

Figure S10. HRESIMS spectrum of **2**.

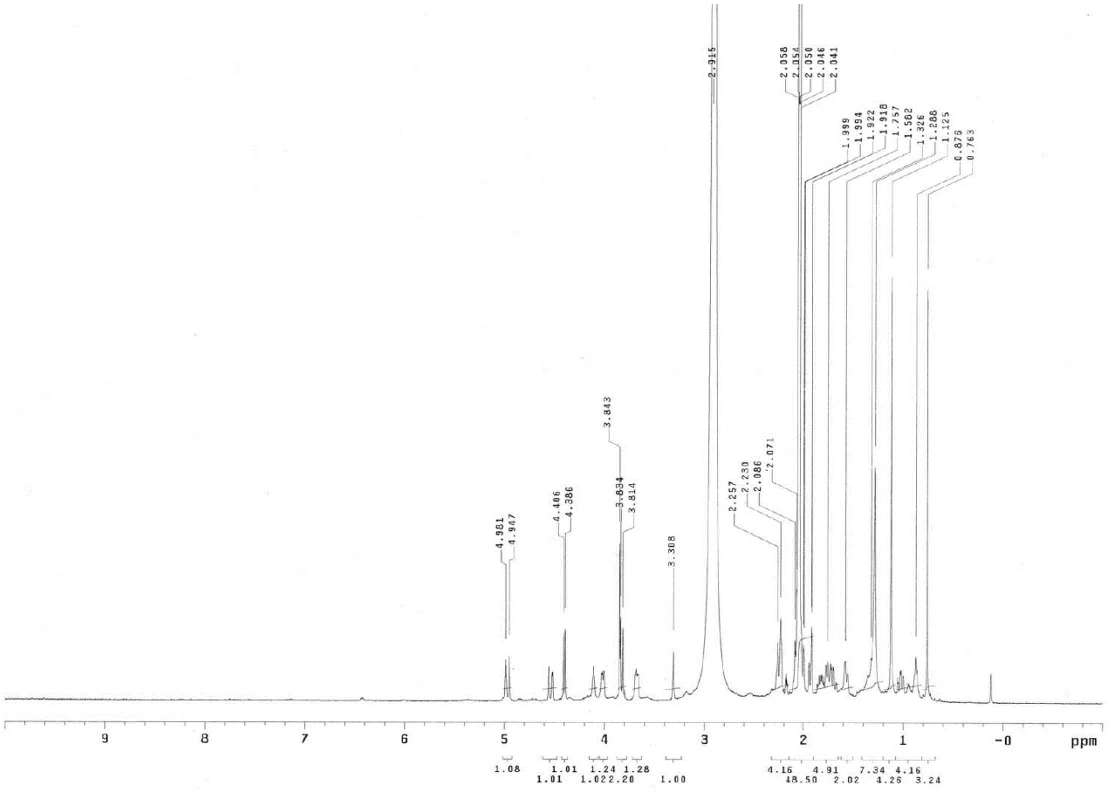

Figure S11. ^{1}H NMR spectrum of **2**.

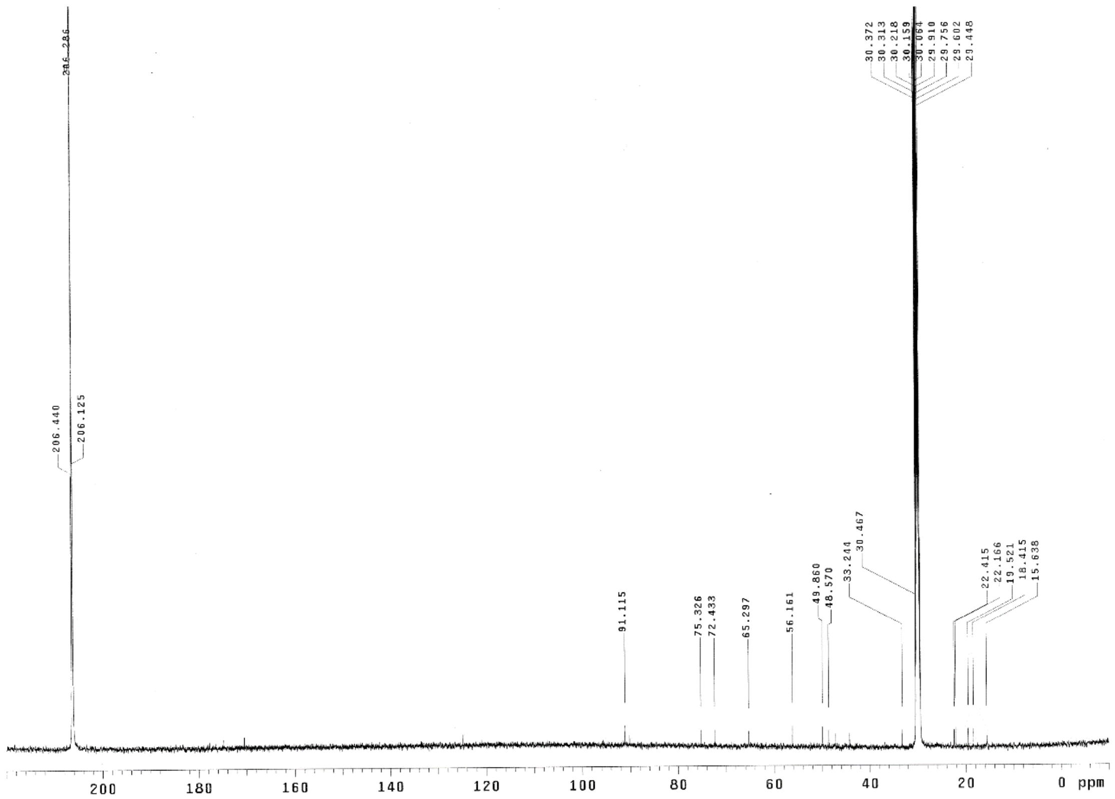

Figure S12. ^{13}C NMR spectrum of **2**.

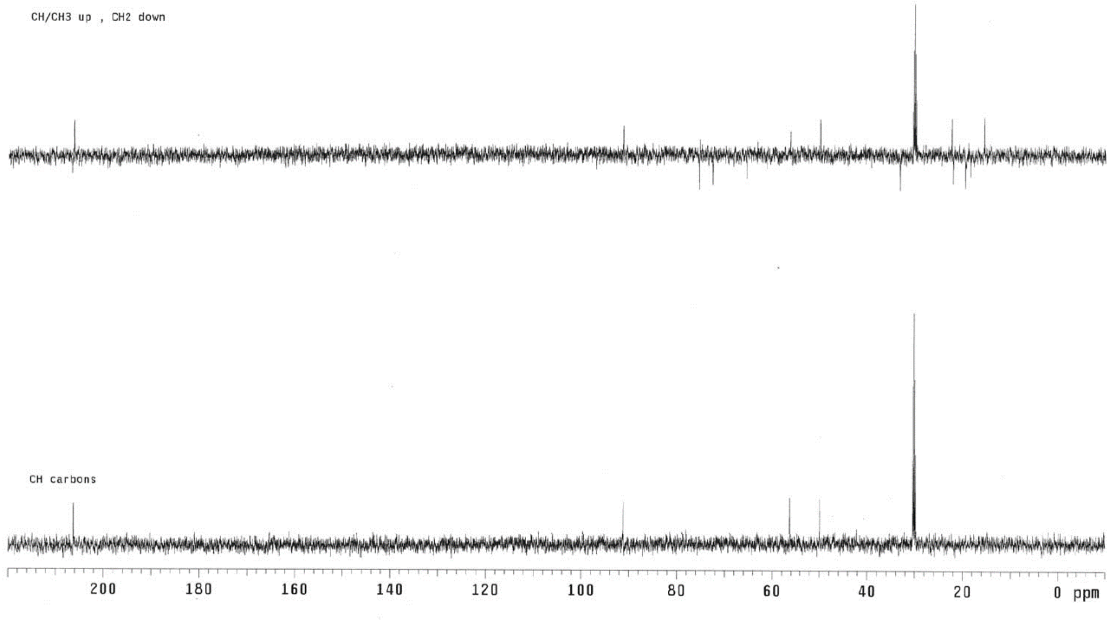

Figure S13. DEPT spectrum of **2**.

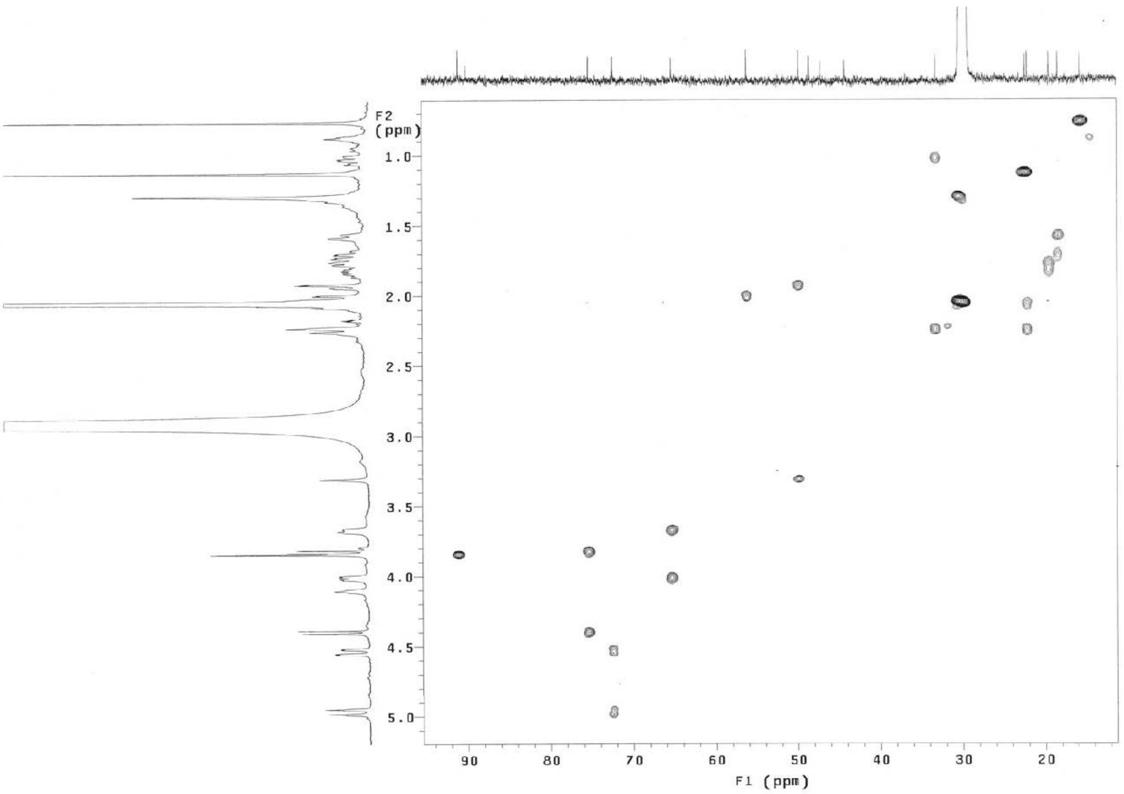

Figure S14. HSQC spectrum of **2**.

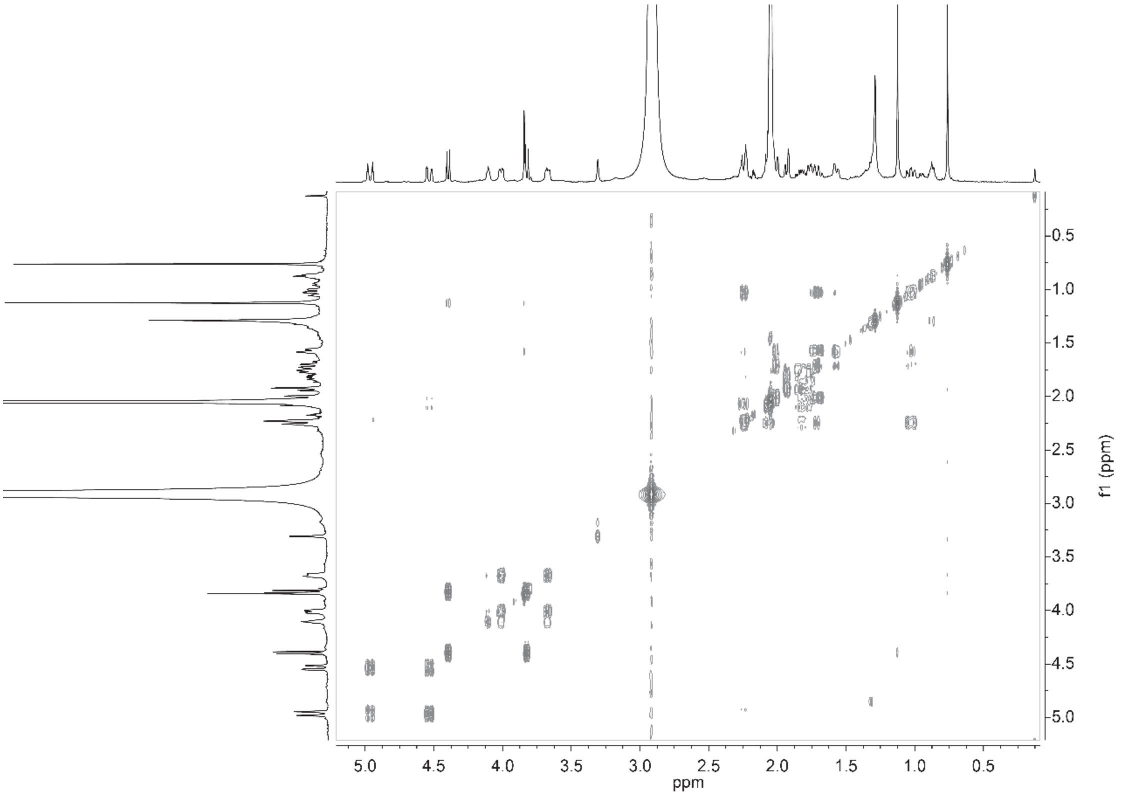

Figure S15. ^{1}H–^{1}H COSY spectrum of **2**.

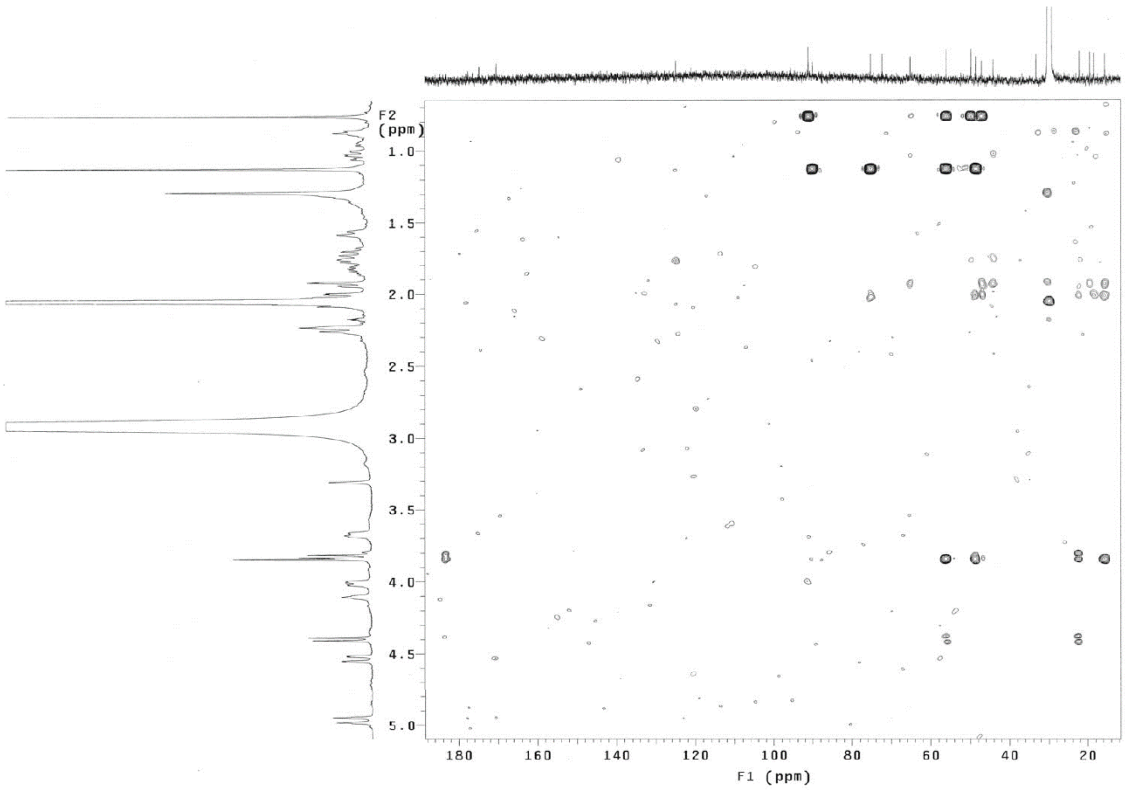

Figure S16. HMBC spectrum of **2**.

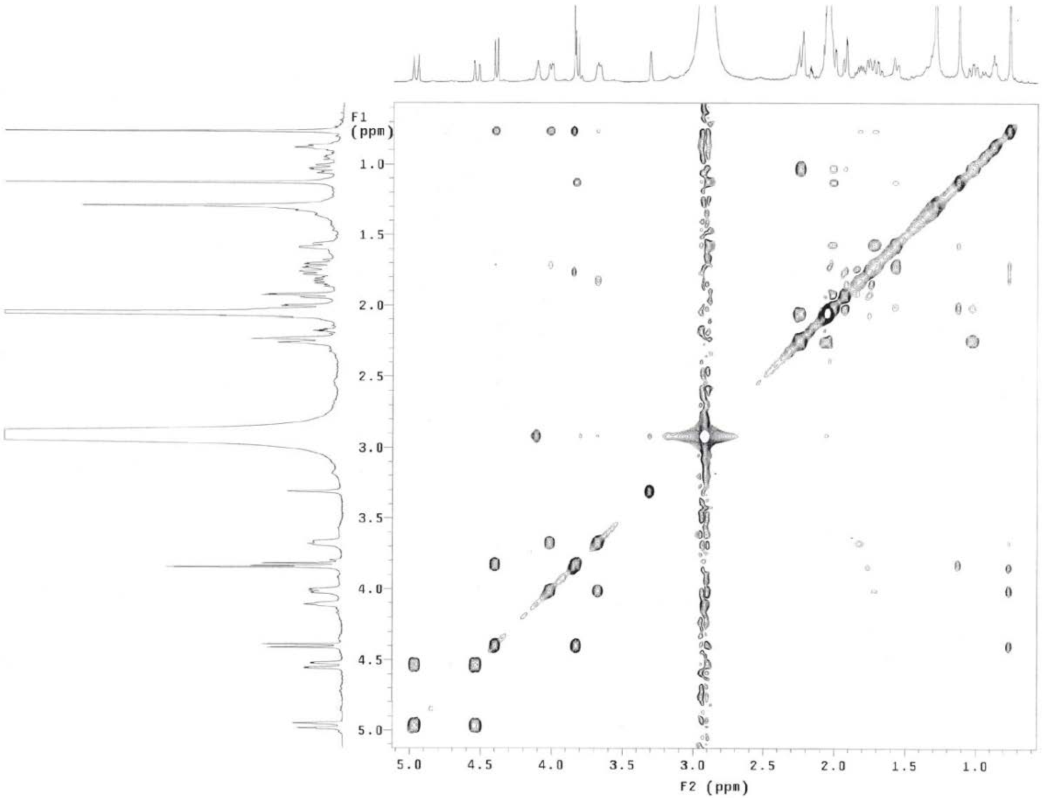

Figure S17. NOESY spectrum of **2**.

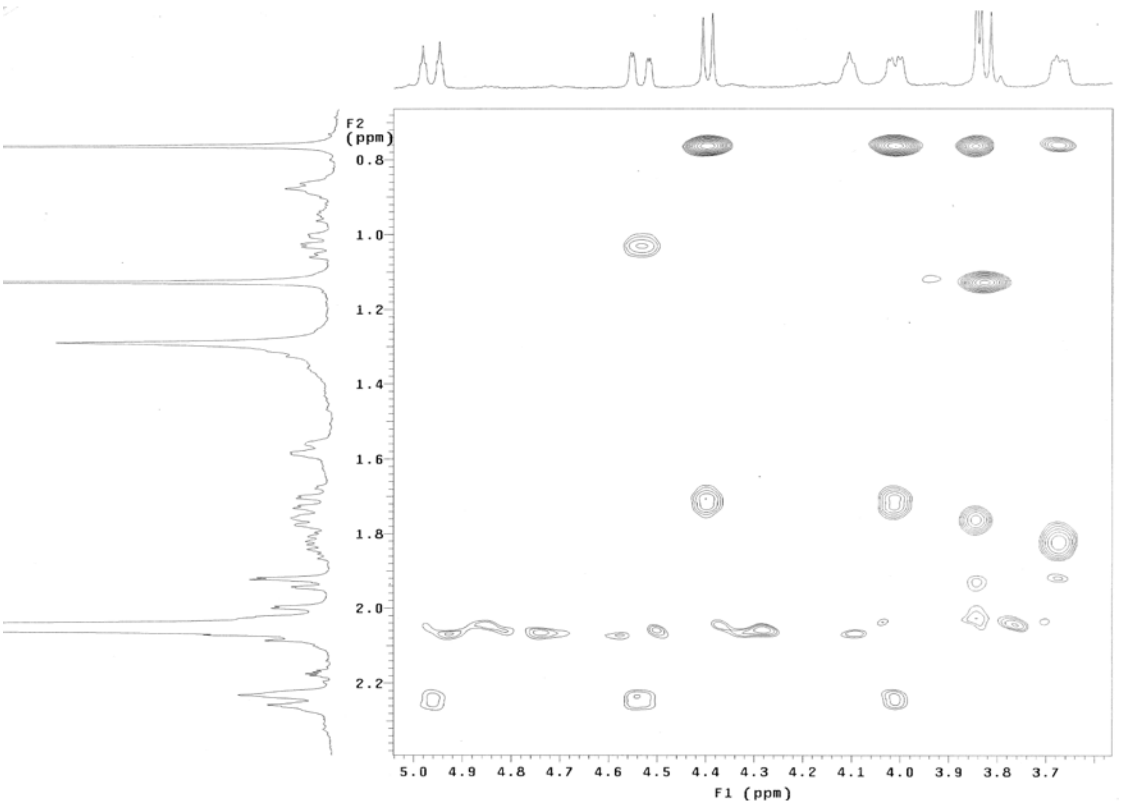

Figure S18. The partial NOESY spectrum of **2**.

Table S2. The cartesian coordinates of conformer **2**.

C	-0.76119200	0.23711000	0.30999000	H	-0.70752100	-0.48368400	-1.67678900
C	-1.12793200	-0.86204000	-0.73168100	H	-0.71223200	-2.92879600	-1.24964800
C	-0.41889900	-2.19300700	-0.48959400	H	-0.66863400	-2.61312300	0.48718400
C	1.10726000	-1.96637100	-0.59624900	H	1.63751100	-2.89825700	-0.36855300
C	1.65734700	-0.81830500	0.30351100	H	1.33535700	-1.71912400	-1.64176600
C	0.76313400	0.48385200	0.14825800	H	0.88333000	0.74808200	-0.91431500
C	3.03470700	-0.37787000	-0.15905600	H	3.30308500	2.66345100	1.07921900
C	3.49152900	0.88463600	-0.10006500	H	2.57955600	2.75594700	-0.51248900
C	2.72052800	2.08764400	0.34931400	H	0.65964300	2.52148500	0.88132500
C	1.35116100	1.67592700	0.93120200	H	1.46007500	1.43238000	1.99413500
C	4.12501500	-1.25840400	-0.70936800	H	4.45578100	-2.03835600	-0.01436900
O	5.23211400	-0.37113000	-0.95431200	H	3.85227600	-1.73818300	-1.65581200
C	4.87587300	0.90744600	-0.60535600	H	-0.57986700	-0.88317000	2.18543100
C	-1.13998500	-0.04151900	1.77752900	H	-0.92463300	0.84532200	2.38345100
C	1.91274700	-1.34049300	1.75756400	H	-2.19979600	-0.26681200	1.91731200
O	5.64477300	1.83966900	-0.72651500	H	2.91690600	-1.78490300	1.78907100
C	-1.64352300	1.38176800	-0.26730700	H	1.91625700	-0.49900600	2.46112800
C	-2.98978300	0.69691300	-0.65779200	H	-1.20107800	1.70882600	-1.21722600
C	-2.66424800	-0.78987700	-0.98914800	H	-4.27257400	-2.23149900	-0.49487100
C	-3.97912700	0.62211700	0.51155400	H	-2.96915800	-2.19741900	0.71154600
O	-4.31123500	-0.64165800	0.80247600	H	-2.55168900	2.44099100	1.11350300
C	-3.54583500	-1.59188400	0.00987400	H	-3.76496000	2.29942400	-1.42104200
O	-1.80863300	2.56662200	0.49579700	H	-2.46992600	-0.58006600	-3.15288800
O	-3.64293200	1.38076200	-1.72009700	H	-2.74051100	-2.24344000	-2.59796100
O	-4.44615200	1.57506500	1.10815700	H	-4.08229700	-1.08426700	-2.62412200
C	-3.01131800	-1.19510800	-2.42852900	H	1.21019300	-2.61072500	3.05454300
O	0.96833400	-2.33311500	2.15650900				

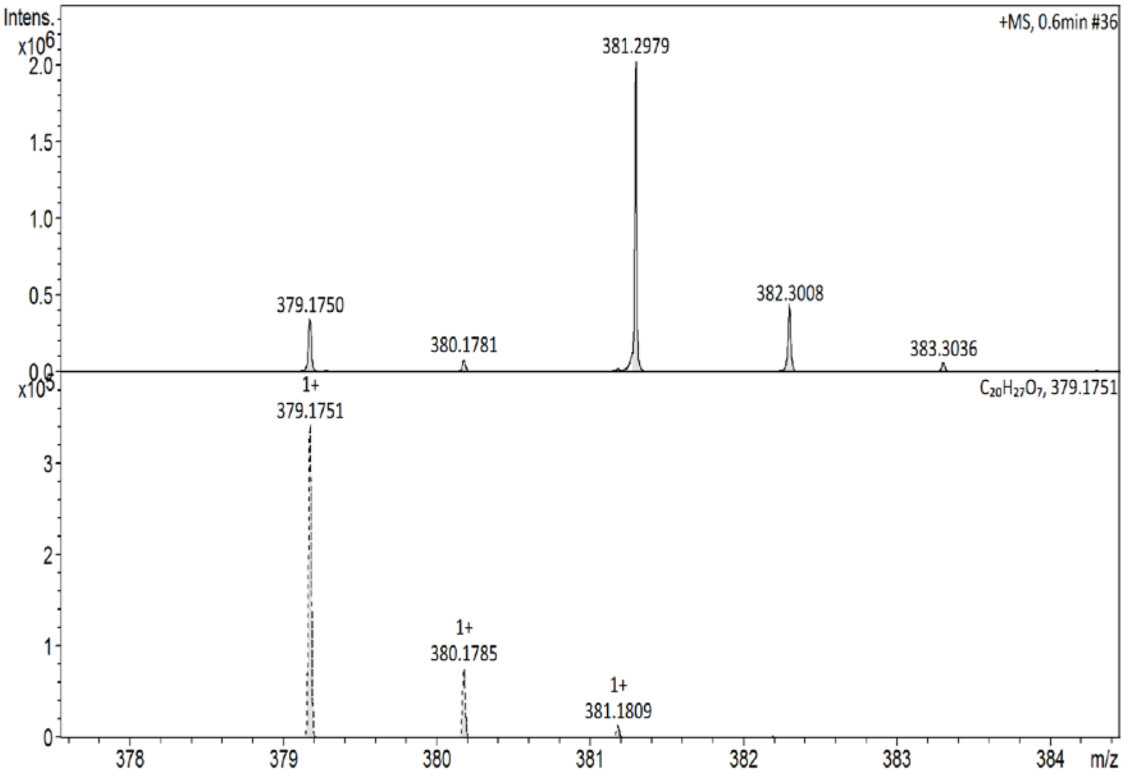

Figure S19. HRESIMS spectrum of **3**.

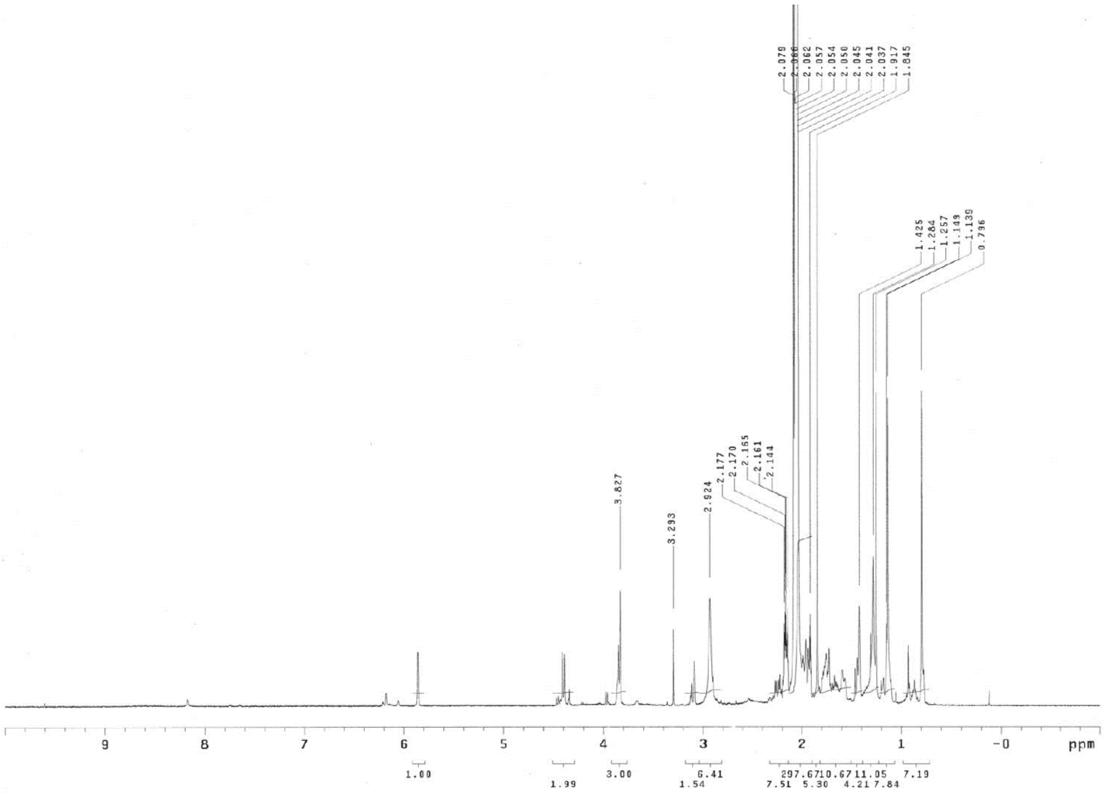

Figure S20. ^{1}H NMR spectrum of **3**.

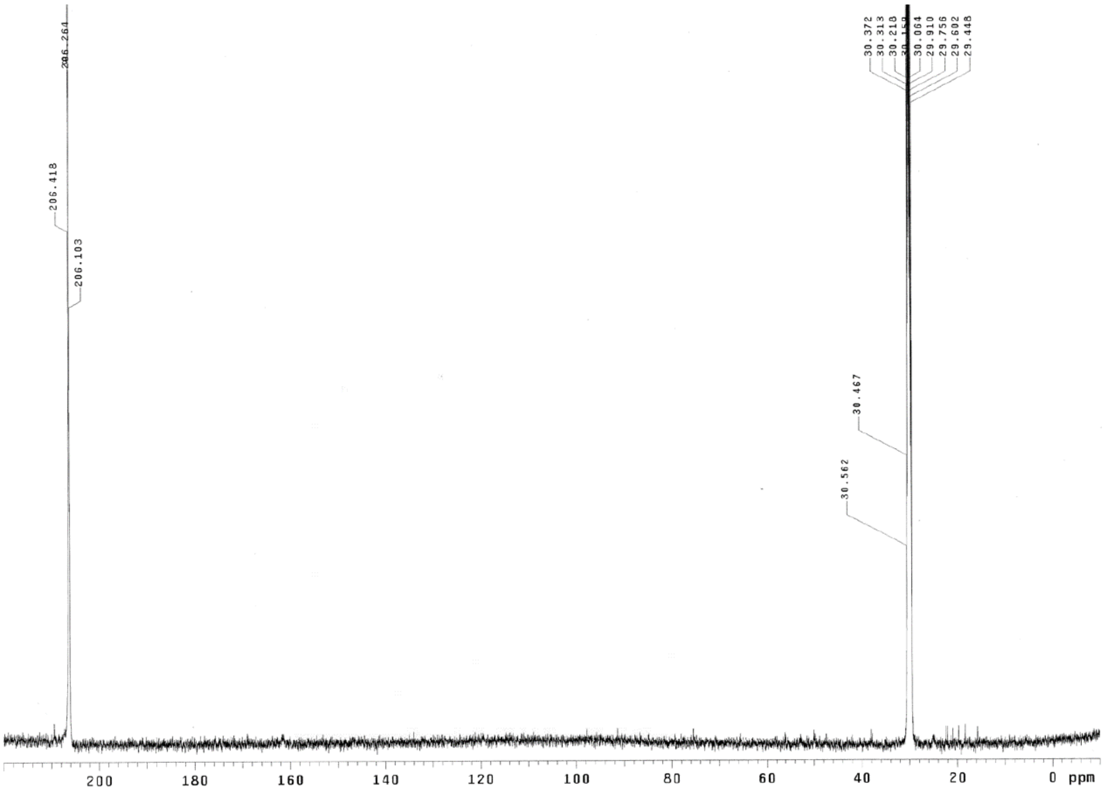

Figure S21. ^{13}C NMR spectrum of **3**.

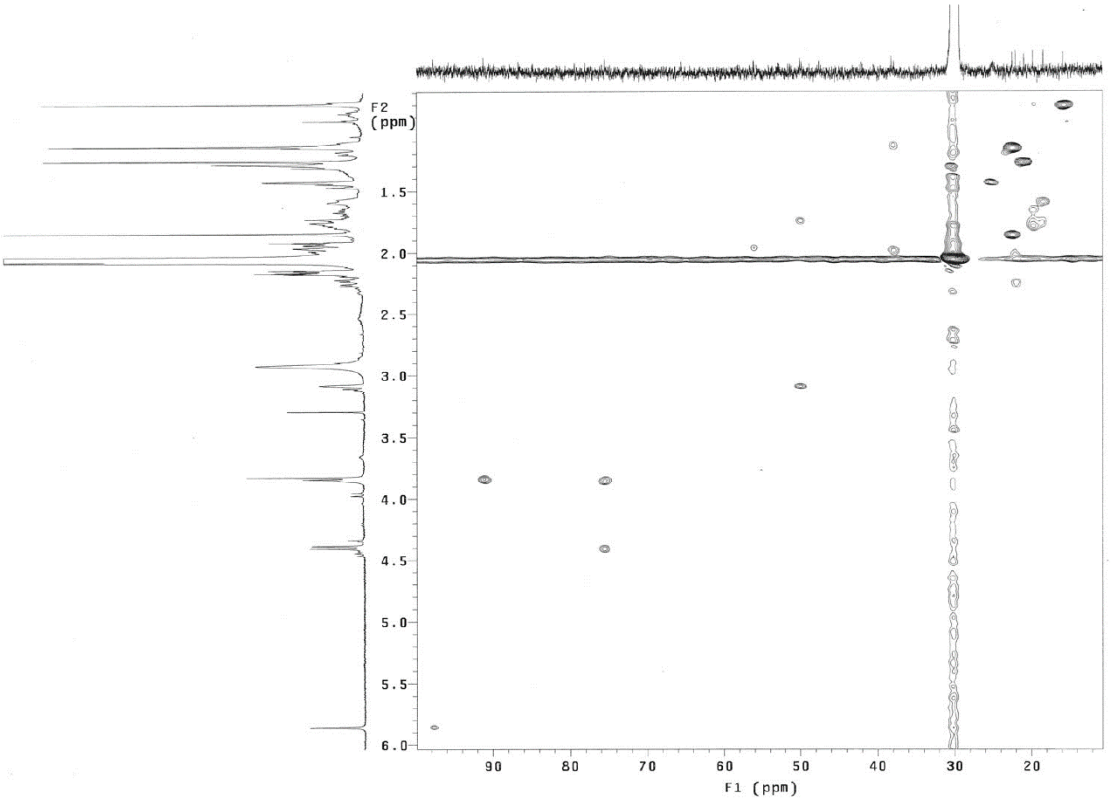

Figure S22. HSQC spectrum of **3**.

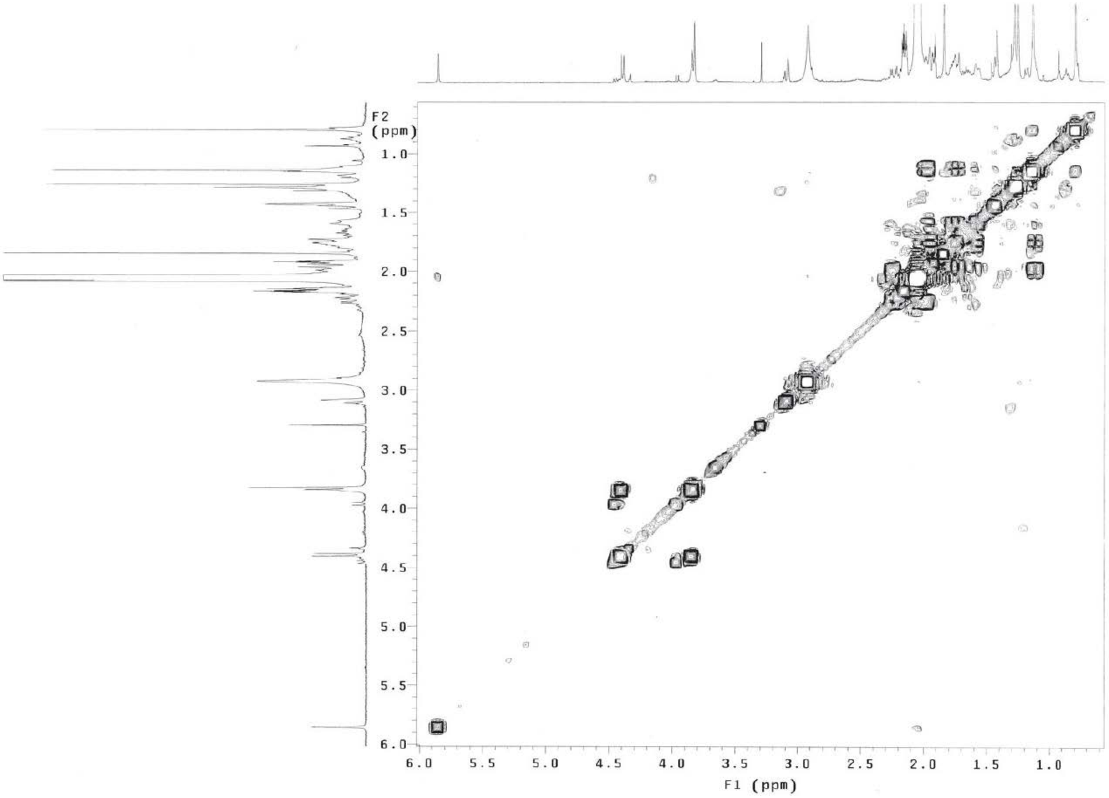

Figure S23. ^{1}H–^{1}H COSY spectrum of **3**.

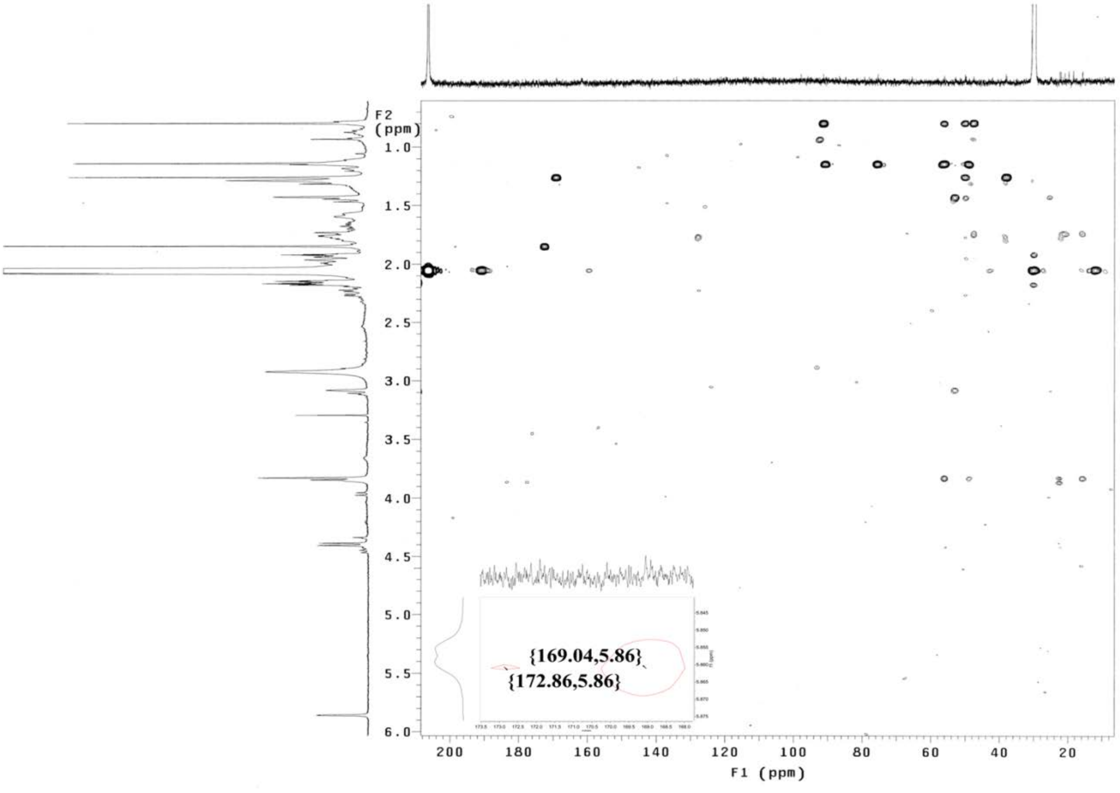

Figure S24. HMBC spectrum of **3**.

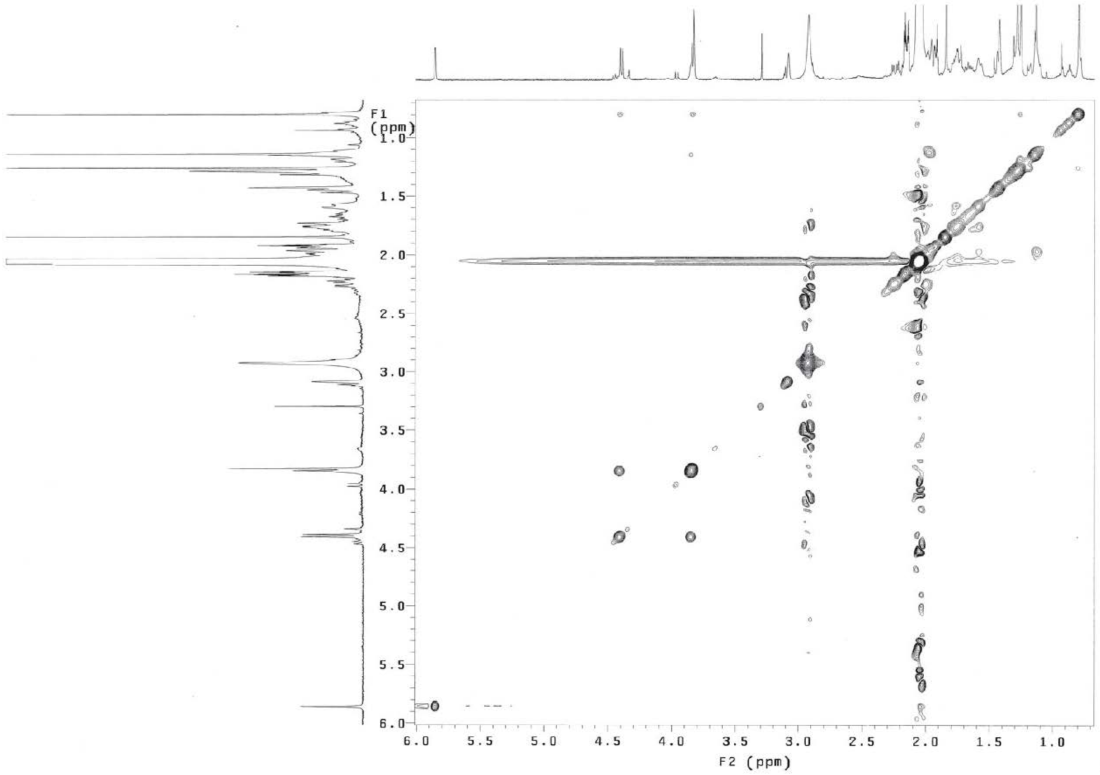

Figure S25. NOESY spectrum of **3**.

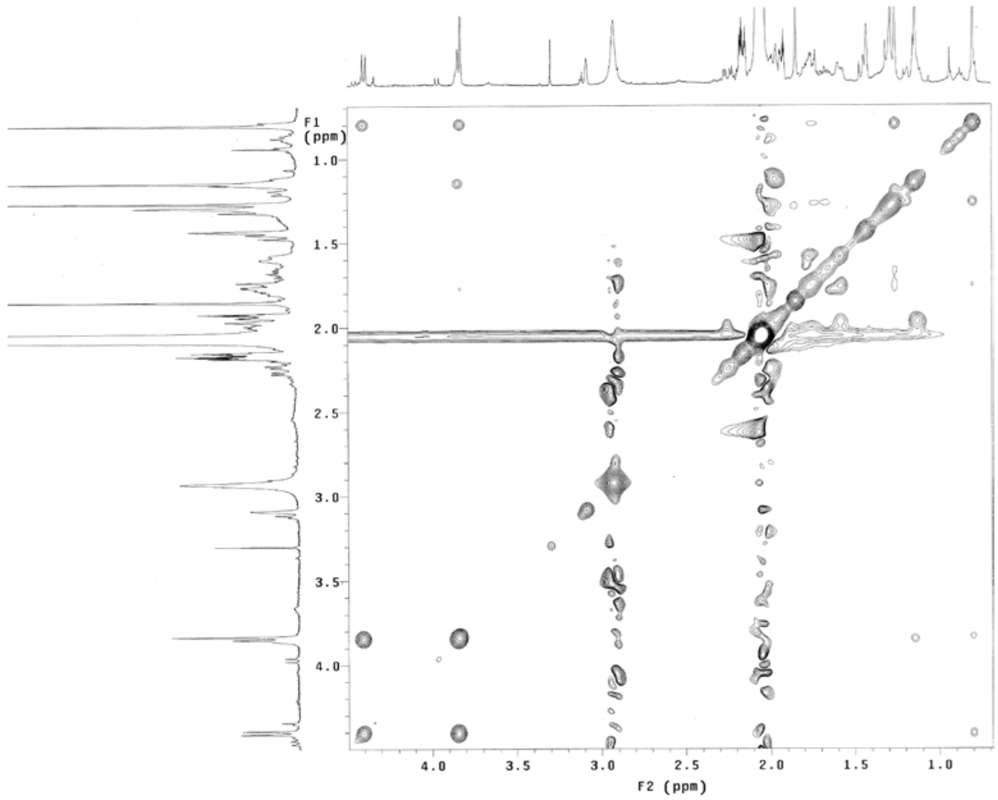

Figure S26. The partial NOESY spectrum of **3**.

Table S3. The cartesian coordinates of conformer **3**.

C	-0.89718400	0.24914900	0.36747300	H	-0.85292400	-0.79890800	-1.47178300
C	-1.22144500	-1.02986700	-0.45948300	H	-0.62406200	-2.52751400	1.01853200
C	-0.42117000	-2.25762100	-0.02477500	H	-0.69300900	-3.13190200	-0.62864700
C	1.07952800	-1.95976800	-0.23612900	H	1.25052100	-1.85337300	-1.31628900
C	1.56492100	-0.66878600	0.48609600	H	1.68597300	-2.81018000	0.09730900
C	0.61294500	0.54241300	0.13053200	H	0.70099000	0.63961200	-0.96318400
C	2.93302600	-0.24935300	-0.01241700	H	2.33108100	2.74566000	-0.90478400
C	3.32932000	1.02219300	-0.17233400	H	3.04374000	2.96566000	0.67933800
C	2.49619700	2.24442800	0.06003000	H	1.27116900	1.79973900	1.79310100
C	1.14598300	1.86993700	0.70704300	H	0.42018500	2.66930600	0.53079000
C	4.06713900	-1.16750700	-0.41334100	H	3.83685900	-1.75342600	-1.31080300
O	5.14343400	-0.27042800	-0.79423400	H	-2.34383000	0.28952700	2.03497900
C	4.72363900	1.02780600	-0.66048700	H	-0.80844400	1.03013100	2.40159700
C	-1.26938900	0.19604800	1.86706200	H	-0.94469800	-0.72610600	2.34956200
C	1.72880000	-0.97513200	2.00342500	H	1.86300600	-0.06597200	2.59582900
O	5.44175900	1.96610500	-0.93356600	H	2.61309300	-1.60077500	2.15007400
C	-1.83999900	1.24964100	-0.35641400	H	0.87296200	-1.52071800	2.40493000
C	-3.13610000	0.43381600	-0.71230100	H	-1.40287200	1.50477300	-1.33403400
C	-2.75878800	-1.07946400	-0.65898900	H	-2.96139800	-1.89636600	1.41078000
C	-4.23549100	0.59627000	0.33137700	H	-4.22775400	-2.43988200	0.29189000
O	-4.45237700	-0.53188800	1.01241800	H	-3.02301300	2.66016100	0.34209400
C	-3.57567400	-1.60601500	0.55799200	H	-4.15808600	1.65952700	-1.82677300
O	-2.07462000	2.44343800	0.37753900	H	-4.23416600	-1.80855600	-2.09344800
O	-3.67011800	0.82894400	-1.96977000	H	-2.87613800	-2.91236900	-1.81321100
O	-4.86765400	1.62085500	0.51413300	H	-2.65281700	-1.45828900	-2.80599500
C	-3.15466500	-1.85803500	-1.92062600	H	5.04276400	-2.68252400	0.28872600
O	4.45974000	-1.98929700	0.64060700				

Table S4. The cartesian coordinates of conformer **3c**.

C	0.89862700	-0.32488400	0.33256800
C	1.21724500	1.06925100	-0.28340700
C	0.45656700	2.21372800	0.38356300
C	-1.05450400	1.98111800	0.16311000
C	-1.55039800	0.59505100	0.67184700
C	-0.62496900	-0.55925500	0.10277000
C	-2.94667800	0.26396300	0.17121600
C	-3.36361600	-0.97266000	-0.15237200
C	-2.52700500	-2.21375500	-0.17779700
C	-1.15723100	-1.95671500	0.48129400
C	-4.12369600	1.21482500	0.06287100
O	-5.23980900	0.36631600	-0.33175300
C	-4.80110200	-0.92019400	-0.48233900
C	1.31324300	-0.53463400	1.80579900
C	-1.67559600	0.65765600	2.22463600
O	-5.55238000	-1.81724200	-0.81648200
C	1.80658700	-1.18184200	-0.59779200
C	3.12524400	-0.35421500	-0.77030500
C	2.74955000	1.14387800	-0.54234800
C	4.18375100	-0.65034800	0.30295700
O	4.46658200	0.43270300	1.03299700
C	3.60626200	1.55841000	0.68682700
O	2.01655200	-2.54860000	-0.26473200
O	3.72938100	-0.58916400	-2.03821900
O	4.73730300	-1.72175200	0.47934200
C	3.07697000	2.05912600	-1.73134300
O	-3.90408600	2.18870000	-0.91133600
H	0.80085700	1.01490800	-1.30136600
H	0.69338400	2.29455900	1.45151800
H	0.72895000	3.17602800	-0.06774500
H	-1.25876200	2.06645700	-0.91058900
H	-1.63095900	2.77549100	0.65099800
H	-0.74940900	-0.48237300	-0.98939400
H	-2.39307700	-2.53065300	-1.22258900
H	-3.05538200	-3.03643900	0.31998600
H	-1.25266100	-2.05911100	1.56818700
H	-0.44709600	-2.72329700	0.15864000
H	-4.41613000	1.66471900	1.01725000
H	2.39540800	-0.58132900	1.94865500
H	0.91525200	-1.48353000	2.17786000
H	0.95021000	0.25444300	2.46378200
H	-1.81241900	-0.33049000	2.67199000
H	-2.54335500	1.26669600	2.50241900
H	-0.80580600	1.12814600	2.68671100
H	1.34930200	-1.20194600	-1.59443500
H	3.01801300	1.78827600	1.57664000
H	4.27102800	2.39672200	0.47215100
H	2.76733300	-2.63418400	0.34922200
H	3.91323600	-1.54274100	-2.11220000
H	4.15005700	2.06341400	-1.94777100
H	2.77456200	3.08750500	-1.50149100
H	2.54872000	1.74100400	-2.63473300
H	-4.59511700	2.86901700	-0.84276100

Table S5. Cytotoxicity (ED_{50} μg/mL) of compounds **1**−**3**.

**Compound**	**ED_{50} (μg/mL)**
	**HCC Huh7 cell**
**1**	＞20 μg/mL
**2**	＞20 μg/mL
**3**	＞20 μg/mL

Results are presented as mean ± SD. (n = 3).
